# Commonly encountered symptoms and their management in patients with cirrhosis

**DOI:** 10.3389/fmed.2024.1442525

**Published:** 2024-11-14

**Authors:** Cyriac Abby Philips

**Affiliations:** Department of Clinical and Translational Hepatology, The Liver Institute, Center of Excellence in Gastrointestinal Sciences, Rajagiri Hospital, Kochi, India

**Keywords:** muscle cramps, insomnia, pruritus, depression, portal hypertension, decompensated cirrhosis

## Abstract

This exhaustive review, explored the multifaceted symptoms and their management in patients with cirrhosis. Patients frequently endure pain, muscle cramps, sleep disturbances, psychological distress, and gastrointestinal issues, significantly impairing their quality of life. Pain is prevalent, often requiring analgesics, while muscle cramps affect up to 68% of patients, treated with supplements like zinc and taurine despite limited evidence. Sleep disturbances, including insomnia and excessive daytime sleepiness, afflict up to 80% of patients, managed through lactulose, melatonin, and cognitive behavioral therapies. Gastrointestinal symptoms, affecting 80%, include abdominal pain and bloating, necessitating lifestyle and dietary adjustments. Mental health disorders, such as depression and anxiety, are common, managed with a combination of pharmacotherapy and psychotherapy. Sexual dysfunction, often overlooked, profoundly impacts both men and women, requiring holistic treatment approaches. Pruritus, another distressing symptom, is managed with moisturizers and antihistamines, though many treatments show limited success. Hair loss and skin changes add to the psychological burden, highlighting the need for a comprehensive, multidisciplinary approach. The review underscores the imperative for tailored, compassionate care to enhance patient outcomes and quality of life in cirrhosis.

## 1 Introduction

Cirrhosis represents the advanced stage of chronic inflammatory liver damage, marked by the disruption of intrahepatic vasculature, hepatocyte loss, and extensive scarring. This leads to the formation of regenerative hepatic nodules encased in dense fibrous tissue, containing significantly higher levels of extracellular matrix proteins, particularly collagen types I, III, and IV, which hinder portal blood flow and elevate portal pressure, ultimately culminating in clinically significant portal hypertension in later disease stages (1). Progression through various disease stages in cirrhosis is fraught with complications related to portal hypertension and chronic liver failure, such as acute variceal bleeding, ascites, hydrothorax, hepatic encephalopathy (HE), life-threatening infections, and extrahepatic organ failure, such as hepatorenal syndrome and additionally, hepatocellular carcinoma (2). Although the management of these complications is well-delineated in clinical guidelines based on robust interventional trial data and are major reasons for hospitalizations, certain symptoms significantly impacting quality of life often prompt recurrent outpatient and emergency visits among individuals with cirrhosis. These encompass a spectrum of maladies including malaise, fatigue and lethargy, appetite disturbances, non-cholestatic pruritus, muscle cramps, sleep disturbances, mental health disorders, gastrointestinal symptoms, sexual dysfunction, pain and peripheral neurological symptoms, hair loss and self-consciousness inducing skin changes. In general, the most frequently reported symptoms in cirrhosis include: pain (prevalence range 30–79%), breathlessness (20–88%), muscle cramps (56–68%), sleep disturbance (insomnia 26–77%, daytime sleepiness 29.5–71%), psychological symptoms (depression 4.5–64%, anxiety 14–45%), and erectile dysfunction (53–93%) in men (3, 4). Regrettably, these symptoms, which exact a financial and resource burden on both patients and healthcare systems, are not currently addressed in guideline-based treatment recommendations.

**Figure 1:**
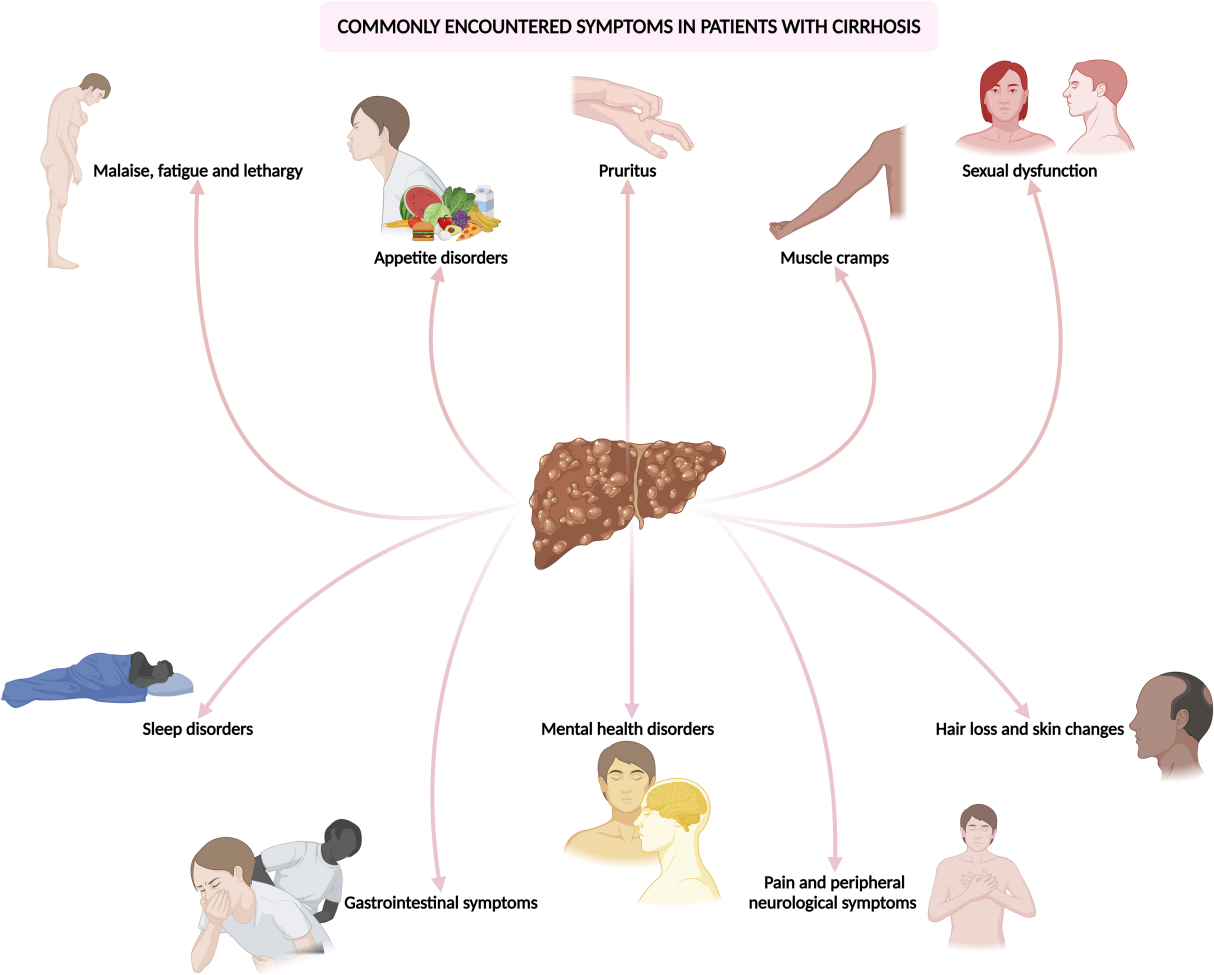
Common symptoms encountered in the outpatient and emergency department in patients with cirrhosis.

In this comprehensive and evidence-driven review, the objective is to scrutinize the repercussions, diagnosis, and management of these often overlooked yet prevalent and challenging-to-treat symptoms encountered in cirrhosis (Figure 1). By disseminating current best practices, the review endeavors to equip physicians caring for patients with liver disease with the requisite knowledge to optimize patient care.

## 2 Cirrhosis-related malaise, fatigue, or lethargy

### 2.1 Definitions and prevalence

Malaise embodies a pervasive sense of discomfort, weakness, or unease, the precise origin of which proves elusive and closely intertwines with mental well-being (5). Conversely, fatigue denotes an overwhelming sensation of tiredness and depleted energy levels that hinder the individual’s customary activities (6). However, lethargy constitutes a more profound manifestation of illness-associated symptoms, characterized by profound drowsiness, extreme lethargy, and cognitive dullness (7). Individuals afflicted with cirrhosis commonly exhibit one or a combination of these symptoms, collectively termed as the non-specific symptom complex or “sickness behaviors,” significantly impacting their quality of life, daily functioning, mental well-being, and productivity. The prevalence of fatigue in cirrhosis exhibits considerable variability across studies, populations, and etiologies, with the definitive prevalence of this condition remain uncertain. Nonetheless, reported prevalence rates range from approximately 60 to 80% in various investigations (4, 7, 8). In contrast, the prevalence of fatigue stands at around 5% in the general population and approximately 20% in non-cirrhotic community settings (9). Although fatigue prevalence fluctuates concerning cirrhosis etiology, ranging from 44% in autoimmune liver disease to as high as 80% in primary biliary cholangitis (7), it is noteworthy that this symptom may not necessarily correlate with the severity of underlying liver fibrosis, advanced disease stages, or organ dysfunction.

### 2.2 Mechanism and causes

Muscle functionality and contractility can suffer impairment either at or beyond the neuromuscular junction, termed peripheral fatigue, or proximal to it, referred to as central fatigue. Notably, fatigue observed in cirrhosis predominantly aligns with the central type. The pathophysiology is intricate, multifaceted, and consequently poorly elucidated. Disrupted peripheral neurogenic signaling pathways between the liver and the brain axis, coupled with perturbed central neurotransmission within the brain, in tandem with disease-related or disease-associated factors, such as complications, treatments, and severity, as well as alcohol use, metabolic syndrome, and malignancy, collectively contribute toward clinical fatigue (10). The interplay of local (within the diseased liver) and systemic inflammation, stemming from portal hypertension and alterations in the gut microbiome, alongside heightened levels of circulating cytokines, disrupts hepatic vagal neurogenic responses and impacts cerebral endothelial cells, giving rise to neuroinflammation. This intricate nexus, predominantly involving peripherally activated immune cells, notably circulating monocytes, and microglia, along with its repercussions on signaling pathways like serotonin, dopamine, and corticotropin-releasing hormone, with the latter’s implications on the hypothalamus-pituitary–adrenal axis, initiates sickness behaviors, including fatigue (4, 8). Moreover, the presence of diminished muscle mass and power, aberrant muscle quality characterized by increased fat or myosteatosis, electrolyte imbalances, malnutrition, subclinical myopathy, diminished motivation, memory, and concentration, alongside an array of mental health and sleep disorders directly linked to or associated with the underlying disease, further exacerbate, and contribute to fatigue in cirrhosis (9).

### 2.3 Diagnosis and management

Peripheral fatigue can be quantified through objective assessments of strength and aerobic capacity, while central fatigue is evaluated using self-reports and validated questionnaires that capture the patient’s perception of physical and mental exertion impacts on their activities and exertion levels. Peripheral fatigue can be measured with tests such as hand-grip strength, short physical performance battery, cardiopulmonary exercise testing, and the 6-min walk test. Central fatigue, on the other hand, is assessed using tools like the visual analog scale, patient-reported outcomes measure information system for fatigue, fatigue severity scale, and fatigue assessment scale (8–10). Diagnosing pathological fatigue necessitates the significant presence of fatigue, reduced energy, and a disproportionate need for rest relative to activity levels. Additionally, at least five ancillary symptoms must be present, such as limb heaviness or generalized weakness, diminished concentration, or attention, decreased motivation or interest in usual activities, insomnia or hypersomnia, nonrestorative sleep, perceived need to struggle against inactivity, pronounced emotional response to fatigue, perceived short-term memory issues, or prolonged malaise following exertion (11).

Managing fatigue in cirrhosis encompasses both non-pharmacological and pharmacological strategies. Non-pharmacological approaches involve addressing the underlying causes of cirrhosis and adhering to the validated TrACE (Treating causes, Ameliorating modifiable factors, Coping mechanisms, and Empathizing) model, particularly used to manage fatigue in primary biliary cholangitis. This model emphasizes treating causes such as electrolyte imbalances, various types of anemia (including screening for testosterone deficiency related anemia), and vitamin and mineral deficiencies, as well as managing comorbidities like metabolic syndrome and glucose control. Patients are educated on avoiding triggers such as shift work, alcohol, tobacco, prolonged bed rest, weight gain, lack of sleep, and stress, while promoting strategies to manage fatigue including increasing exercise, consuming nocturnal protein snacks, and employing ergogenic nutrition (2, 4, 9).

Pharmacological treatments, however, have shown limited evidence of effectiveness in alleviating fatigue. Several drugs have been studied, but none have demonstrated significant benefits, indicating a need for larger, well-designed, controlled trials. These drugs include ondansetron (small benefit in a small randomized trial), S-adenosyl-methionine (SAMe, small benefit in uncontrolled studies), pentoxifylline (no benefit), modafinil (no benefit), hydroxyzine, zolpidem, trazodone, and melatonin (no benefit for sleep quality and insomnia) (8, 9).

**Figure 2:**
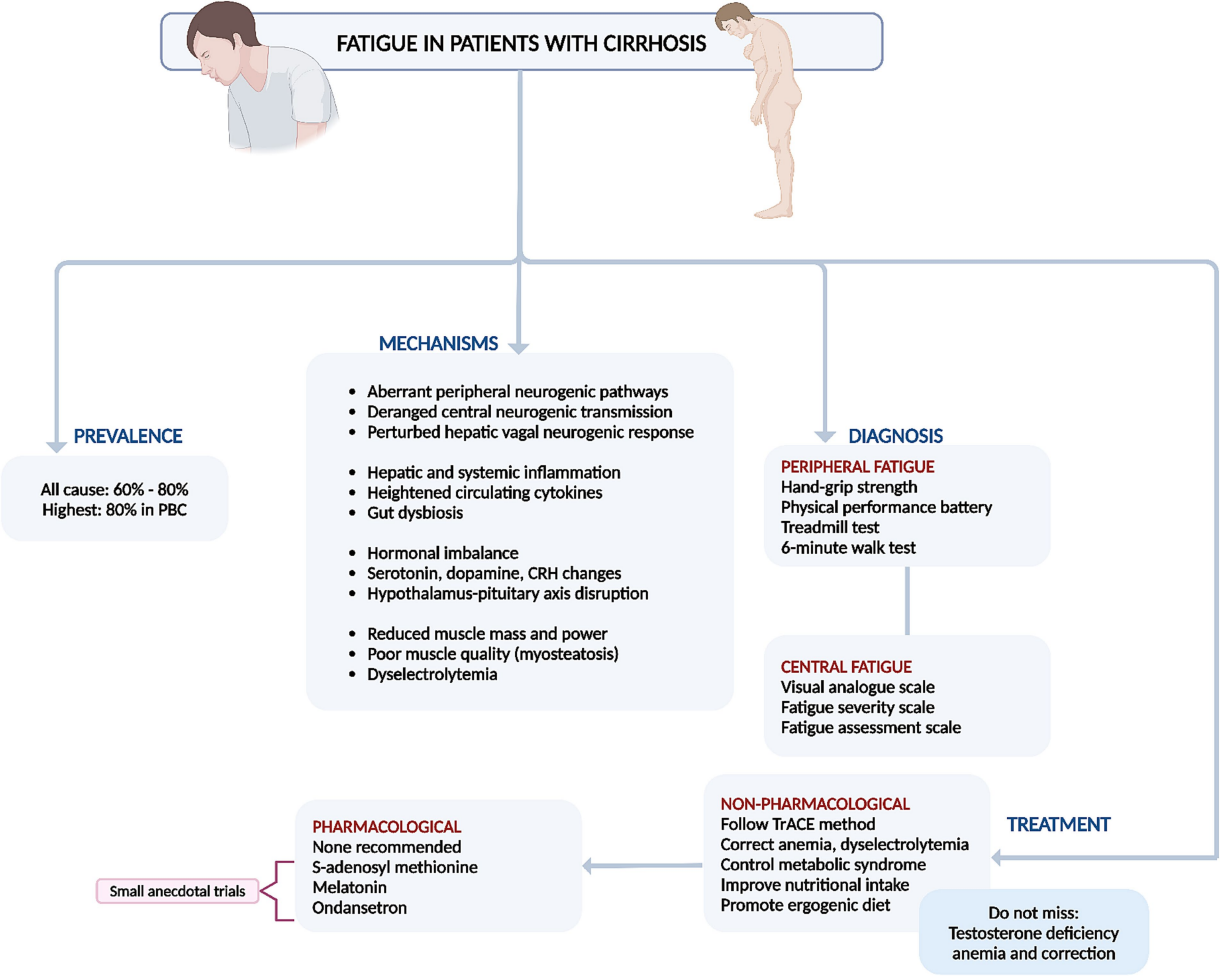
Summary of prevalence, mechanisms, diagnosis, and management of fatigue in cirrhosis. PBC, primary biliary cholangitis.

In conclusion, non-pharmacological therapies that focus on controlling the etiology of liver disease, improving nutrition, increasing muscle mass, mitigating triggers and risk factors, and enhancing coping mechanisms are significantly more effective in combating fatigue in cirrhosis compared to pharmacological treatments (Figure 2) (4, 9).

## 3 Cirrhosis-related appetite disorders, restrictive diet, and malnutrition

### 3.1 Prevalence, risk factors and mechanisms

Chronic liver failure in end-stage cirrhosis often leads to low appetite, nutritional deficits, and malnutrition. Decompensated cirrhosis patients frequently report low appetite during outpatient visits, with malnutrition affecting 50–90% of those with advanced cirrhosis (12). Appetite issues in cirrhosis, including decreased food intake and quality, stem from various factors. Clinical complications related to liver failure and portal hypertension, such as hepatic encephalopathy, symptomatic ascites, repeated hospitalizations, medication burden, electrolyte imbalances (especially hyponatremia), low sodium diets, and general debility, exacerbate appetite loss (13). A small study found that over one-third of cirrhosis patients experienced reduced appetite (14). Chemosensory dysfunctions in chronic liver disease patients alter smell and taste, affecting appetite and leading to nutritionally inadequate food cravings and preferences (15). Inflammation-associated anorexia, prevalent gastrointestinal symptoms due to slow intestinal transit, disrupted appetite regulation, and energy expenditure—linked to overexpression of leptin, increased bound leptin, irregular ghrelin secretion, and blunted ghrelin responses—correlate with liver disease severity and systemic inflammation, further reducing food intake quantity and frequency of spontaneous food consumption (12, 16). Additionally, advanced cirrhosis patients often experience abdominal symptoms such as pain and reflux, and are sarcopenic, a factor independently associated with mortality (13). Currently, there are no validated guidelines for assessing appetite disorders in cirrhosis, highlighting an unmet need to identify those at risk of liver-related complications and malnutrition. However, the Simplified Nutritional Appetite Questionnaire (SNAQ) may be useful in identifying patients with poor appetite and at risk of unhealthy weight loss and malnutrition (17).

### 3.2 Treatment

**Figure 3:**
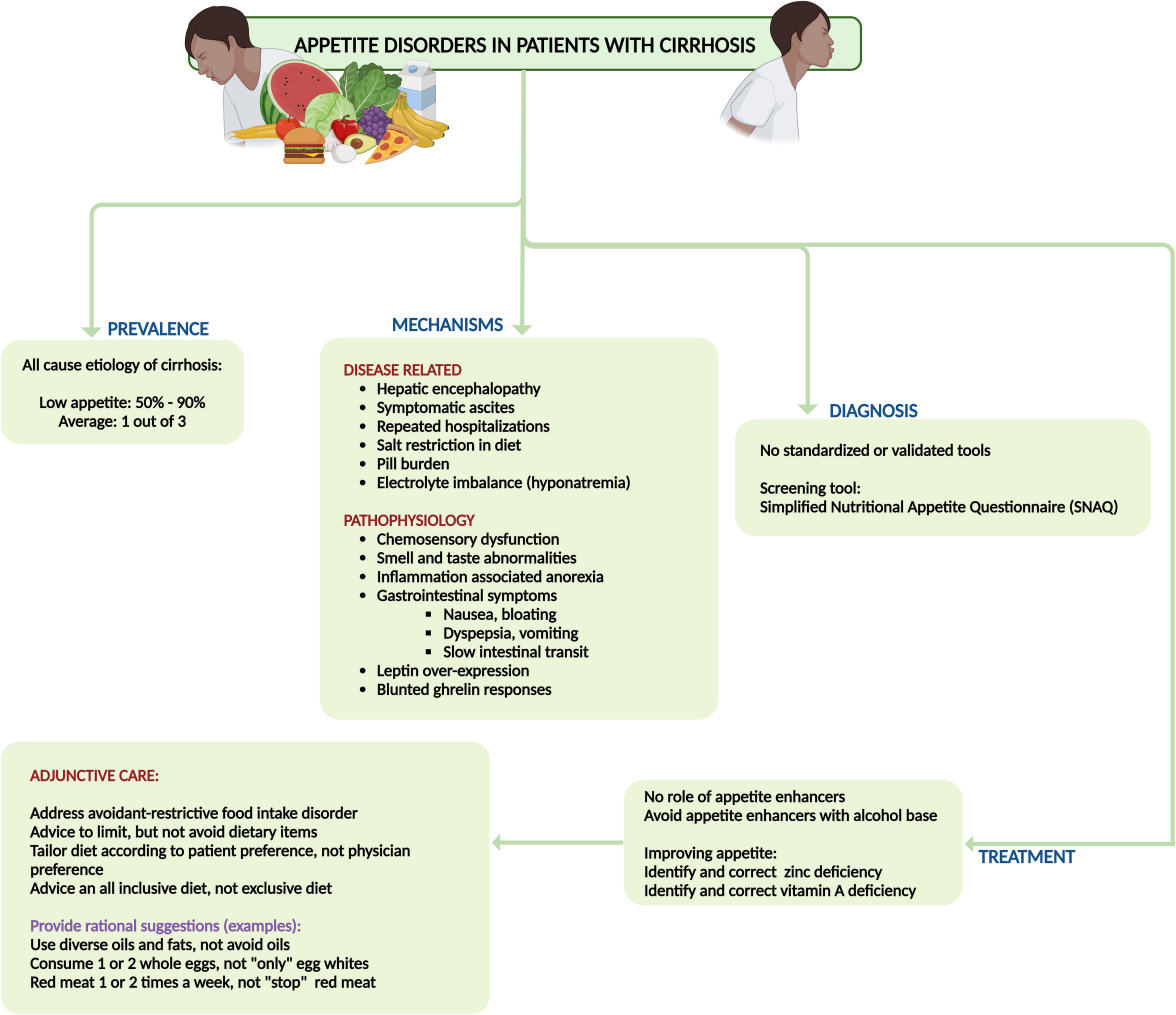
Summary of prevalence, mechanisms, diagnosis, and management of disorders of appetite and malnutrition in cirrhosis.

It is crucial to address the patient holistically by managing risks and triggers, rather than relying only on pharmacological treatments. There is currently no evidence that “appetite enhancers” effectively improve symptoms or increase nutritional intake in cirrhosis patients. Treating low appetite in advanced cirrhosis should focus on addressing risk factors (such as active alcohol use, uncontrolled diabetes, and hypothyroidism), triggers (including hepatic encephalopathy, infections, electrolyte imbalances, and excessive medication burden), and related issues (such as removing symptomatic ascites, providing clear dietary recommendations, avoiding overly restrictive diets, and addressing mental health concerns) (13, 18). International guidelines recommend a sodium intake restriction of 2,000 mg/day for patients with decompensated cirrhosis. However, simplistic advice to “avoid salt” and processed foods, combined with misguided traditional dietary advice (such as avoiding whole eggs, dairy, animal-based proteins, oils, and fats), can lead to avoidant-restrictive food intake disorder (19). This negatively impacts both mental health and appetite due to fearmongering and confusion about nutritional choices. Sodium restriction alone alleviates ascites in only about 10–15% of patients, and some studies show no additional benefit over an unrestricted diet when diuretics are used, if sodium intake adheres to recommended levels. Enhancing food palatability with a diverse, patient-tailored diet that avoids added salt and ensures well-spaced diet intake could help mitigate appetite disorders in cirrhosis (18). Taste changes (dysgeusia), a common issue in cirrhosis, contribute to low appetite. Zinc and vitamin A, which are essential for maintaining taste integrity and often deficient in advanced cirrhosis, should be identified and corrected to improve appetite and food palatability in these patients (Figure 3) (13, 18).

## 4 Cirrhosis-related non-cholestatic pruritus

### 4.1 Prevalence and mechanisms

Pruritus linked to liver ailments is particularly pronounced in chronic cholestatic liver conditions like primary biliary cholangitis and primary sclerosing cholangitis. While the treatment for pruritus stemming from cholestatic liver disorders is established, pruritus originating from non-cholestatic causes is frequently encountered in cirrhotic patients due to various non-cholestatic factors. The prevalence of pruritus varies depending on the underlying cause, with reports indicating rates of up to 70% in primary biliary cholangitis, 2.5–30% in chronic hepatitis C virus-related liver disease, and roughly 8% in cirrhosis attributed to hepatitis B virus infection (20). Pruritus in cirrhosis typically affects larger body areas such as the back and abdomen, sparing smaller extremities like the soles and palms, hands, and feet. It tends to intensify during daytime rather than nighttime, with exacerbations commonly occurring during the winter season and cold climates (20, 21). Factors such as severe thrombocytopenia, serum aspartate aminotransferase levels exceeding 60 U/L and the presence of diabetes have been identified as independent predictors associated with pruritus in cirrhosis (21).

Numerous hypotheses have been posited to elucidate the genesis of pruritus, albeit none deemed definitive. These include heightened endogenous opioid production and the activation of *μ*-opioid receptors, instigating itching by attenuating pain signaling. Additionally, elevated levels of circulating bile acids and dysregulated synthesis of lysophosphatidic acid and autotaxin are implicated. Pruritus characteristic of cirrhosis predominantly manifests as a central phenomenon, purportedly stemming from augmented release of β-endorphin, Met-enkephalin, and endomorphin-1,2, which stimulate *μ*-receptors within nerve tissues. Conversely, in the peripheral variant, histamine discharge from mast cells triggers the activation of itch receptors at the epidermal-dermal interface. The pruritus associated with cirrhosis exacts a toll on quality of life, exacerbates sleep disturbances and mental health disorders including suicidal ideation, and serves as a primary driver of recurrent outpatient consultations.

### 4.2 Diagnosis and treatment

The crucial first step in diagnosing the root cause of itching demands a meticulous investigation into the presence of a rash, a potential pointer to dermatologic cause. Should this prospect be dismissed, an exhaustive exploration of other potential culprits is imperative, including systemic causes (both hepatic and extrahepatic), neurological disorders, and psychogenic triggers (20). The localization of itching implies an origin rooted in either neurological dysfunction or psychogenic factors. A comprehensive medical history is paramount, delving into risk factors for liver disease, existing medical comorbidities, general symptoms, and the utilization of medications or substances. Employing patient questionnaires at the outset and throughout treatment serves as a valuable tool for assessing the gravity of itching and tracking therapeutic responses. Various standardized scales, such as the Numerical Rating Scale, Dermatology Life Quality Index, and 5D Itch Scale, serve as objective metrics in clinical settings, evaluating diverse facets of itching encompassing its intensity, distribution, and associated conditions like anxiety, depression, and insomnia, along with overall quality of life (22). In instances suggestive of biliary obstruction, the deployment of imaging modalities such as ultrasound or cross-sectional imaging is warranted.

**Figure 4:**
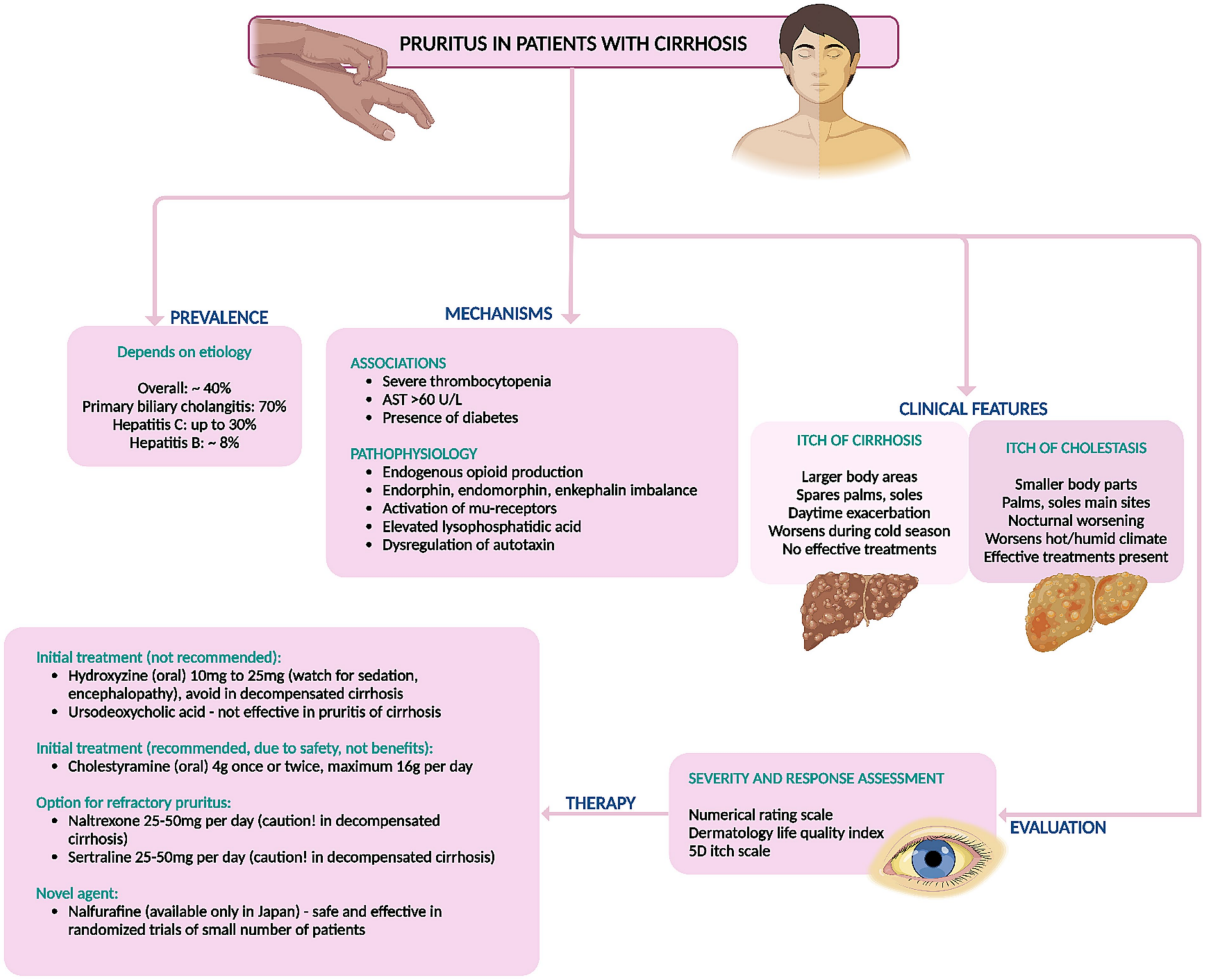
Summary of prevalence, mechanisms, diagnosis, and management of non-cholestatic itching (pruritus) in cirrhosis. AST, aspartate aminotransferase.

There are no recommended pharmacological therapies for control of pruritus in cirrhosis. The armamentarium includes various medications that are tailored toward patient tolerability and considering underlying liver disease severity. Skin changes and temperature dysregulation leading to loss of turgor, dryness and therefore itching and surface breaks are common in cirrhosis and the use of moisturizers to improve hydration and maintain barrier integrity is always the first step toward reducing pruritus (4). Antihistamines like hydroxyzine and diphenhydramine are frequently employed as initial treatments for pruritus due to their safety and availability. However, their efficacy data are limited, and their sedative effects can potentially induce hepatic encephalopathy in patients with chronic liver failure. Furthermore, hydroxyzine can prolong the QT interval, potentially leading to fatal torsades de pointes. It is crucial to recognize that patients with cirrhosis often experience electrolyte imbalances, making them particularly susceptible to QT interval prolongation (4, 8). Ursodeoxycholic acid (UDCA) has been found ineffective for pruritus relief in primary sclerosing cholangitis and primary biliary cholangitis but is recommended for treating pruritus in intrahepatic cholestasis of pregnancy only, at doses of 10–15 mg/kg/day, divided into 2–3 doses. There is no evidence supporting its benefit in non-cholestatic pruritus associated with cirrhosis (4, 20). Cholestyramine, a bile salt resin, effectively alleviates cholestatic pruritus and is the first-line treatment even in cirrhosis due to its general tolerability and safety (4). Potential gastrointestinal side effects include constipation and, rarely, fat malabsorption. Dosages range from 4 g once or twice daily, up to a maximum of 16 g/day in divided doses (4, 8, 20). Other medications should be administered either 1 h before or 4–6 h after cholestyramine intake to avoid interaction. Rifampicin has shown effectiveness in alleviating cholestatic pruritus but poses a hepatotoxicity risk in up to 13% of patients with prolonged use and has not been studied for pruritus in cirrhosis (4, 23). Naltrexone, a *μ*-opioid receptor antagonist, dosed at 25–50 mg daily, has demonstrated efficacy in small trials for treating cholestatic pruritus. However, it is contraindicated in patients with chronic liver failure due to concerns about hepatic encephalopathy and associated symptoms like malaise, nausea, appetite loss, and abdominal cramps (23). Sertraline has shown moderate effectiveness in reducing pruritic symptoms and is generally well-tolerated. However, it should be used cautiously in patients with advanced liver disease and is generally not recommended (8, 23). A 2021 study on nalfurafine, a κ-opioid agonist metabolized by cytochrome P450 into an inactive form, showed significant improvement in pruritus scores without notable adverse effects at doses of 2.5 mg and 5 mg, regardless of daytime or nighttime itching (24). A summary of treatment options for pruritus in cirrhosis is shown in Figure 4.

## 5 Muscle cramps

### 5.1 Prevalence and mechanisms

Muscle cramps are involuntary, at times visible, and painful contractions of skeletal muscles that can occur at rest or be intense enough to awaken a person from sleep. Muscle cramps can endure from a few seconds to several minutes, often causing lasting tenderness and swelling for up to 72 h after an episode. They predominantly occur at night, leading to significant sleep disturbances. Muscle cramps are an independent risk factor for diminished health-related quality of life. They disrupt sleep, hinder physical functioning and mobility, and negatively impact general and mental health (25). The prevalence of muscle cramps in liver cirrhosis varies widely, ranging from 29 to 88% depending on the investigators’ inclusion criteria. Recent studies comparing cirrhosis and chronic hepatitis revealed that muscle cramps were significantly more common in cirrhosis patients (51.8–52.0%) than in those with chronic hepatitis (7.5–43.7%). Muscle cramps predominantly afflict the lower limb muscles, notably the calves and feet, though the fingers and hands may also be susceptible (4, 8, 26). The prevalence of cramps among individuals with chronic liver disease spans from 22 to 88%, mirroring the incidence observed in other chronic conditions such as type 1 diabetes mellitus (24–34%), type 2 diabetes mellitus (45–78%), and chronic kidney disease (56–67%), yet markedly exceeding that of the general populace. Factors such as female sex, concomitant diabetes, and chronic kidney disease were linked with the occurrence of muscle cramps in chronic liver disease. Additionally, diminished muscle mass was associated with muscle cramps in nonalcoholic fatty liver disease (27). These findings indicate that muscle cramps in cirrhosis are independently associated with the severity of the liver disease and declining liver function.

The precise pathophysiology of muscle cramps persists in its obscurity, and efficacious treatments have yet to be ascertained. Researchers discerned no correlation between the incidence of muscle cramps and factors such as edema, ascites, diuretic use, alcohol consumption, but have identified the presence of liver cirrhosis, higher total serum bilirubin levels, and lower serum albumin levels as risk factors for development of muscle cramps (25, 28). The etiology of muscle cramps is complex and multifactorial. Hyperexcitability of motor nerve terminals can be incited by disrupted energy metabolism, ischemic damage resulting from reduced intravascular volume, and electrolyte imbalances. Oxidative stress, along with structural changes such as axonal loss and demyelination from toxic insults like hyperglycemia and alcohol consumption, are also associated factors. In cirrhosis, decreased adenosine triphosphate production in muscles can cause ion channel dysfunction and destabilization of the sarcolemma, leading to prolonged muscle contractions. Studies have revealed no significant differences in electrolyte concentrations, diuretic use, or liver disease severity among patients experiencing muscle cramps. However, shifts in plasma volume play a pivotal role, as the presence of ascites, lower mean arterial pressure, and higher plasma renin activity have been identified as key predictors of muscle cramps (29). Even though severe magnesium deficiency has been unequivocally linked to muscle cramping, as magnesium is believed to mitigate muscular excitability, studies found no correlation between serum magnesium levels and the occurrence of muscle cramps in patients with cirrhosis (25, 28).

### 5.2 Treatment

The therapeutic arsenal for alleviating muscle cramps in cirrhosis spans a variety of agents, including vitamin E, pregabalin, intravenous albumin, eperisone, taurine, zinc, baclofen, methocarbamol, orphenadrine, branched-chain amino acids, L-carnitine, and quinidine. The efficacy of these medications has largely been evaluated through small cohort studies and uncontrolled case series, rendering them less recommended for treating muscle cramps comprehensively (25, 28). Vitamin E (200 mg thrice daily for 4 weeks) demonstrated significant improvement in a limited case series; however, a subsequent double-blinded, placebo-controlled trial failed to replicate these benefits and even noted exacerbation in some instances. L-carnitine, crucial in fatty acid metabolism and often deficient in cirrhosis patients, significantly mitigated cramps at doses ranging from 300 mg twice daily to 1,200 mg/day. Supplementation coupled with exercise proved advantageous, with no adverse events reported, albeit in small, uncontrolled studies. Zinc supplementation (220 mg twice daily for 12 weeks) showed efficacy in a minor study, notwithstanding one case of diarrhea. Notably, zinc levels did not correlate with cramp occurrence. Taurine, a nonessential amino acid, effectively reduced muscle cramps in cirrhosis patients. Studies administering 3 gm/day to 6 gm thrice daily over 1 to 24 months reported significant improvements, corroborated by recent trials with doses up to 2 gm/day without adverse effects. Vitamin D supplementation (0.5–1.0 μg for 2 weeks) reduced cramps in about half of the patients, though no RCTs have been conducted. Branched-chain amino acid levels, typically reduced in cirrhosis patients, were restored through supplementation, which alleviated muscle cramps, particularly with higher and nocturnal doses, yielding minimal self-limiting adverse events. Baclofen, a muscle relaxant, demonstrated substantial reductions in cramp severity and duration, with a small randomized controlled trial confirming its efficacy and tolerability. Pregabalin, for neuropathic pain, significantly decreased cramp frequency versus placebo, though pain intensity during sleep remained unaffected. It was well tolerated. Methocarbamol (500 mg twice daily) reduced cramp frequency and severity with no severe side effects, though benefits diminished post-cessation. Orphenadrine (100 mg twice daily), an anticholinergic, significantly reduced cramp frequency, severity, and duration, as supported by a placebo-controlled trial with minimal side effects. Intravenous albumin lowered cramp frequency in a randomized cross-over trial, warranting further investigation. Quinidine (400 mg/day) reduced cramp incidence by 88% versus 13% with placebo, though some patients experienced diarrhea. Eperisone hydrochloride (150–300 mg/day) also reduced cramp frequency, with common side effects including fatigue and dizziness (25, 28, 29).

**Figure 5:**
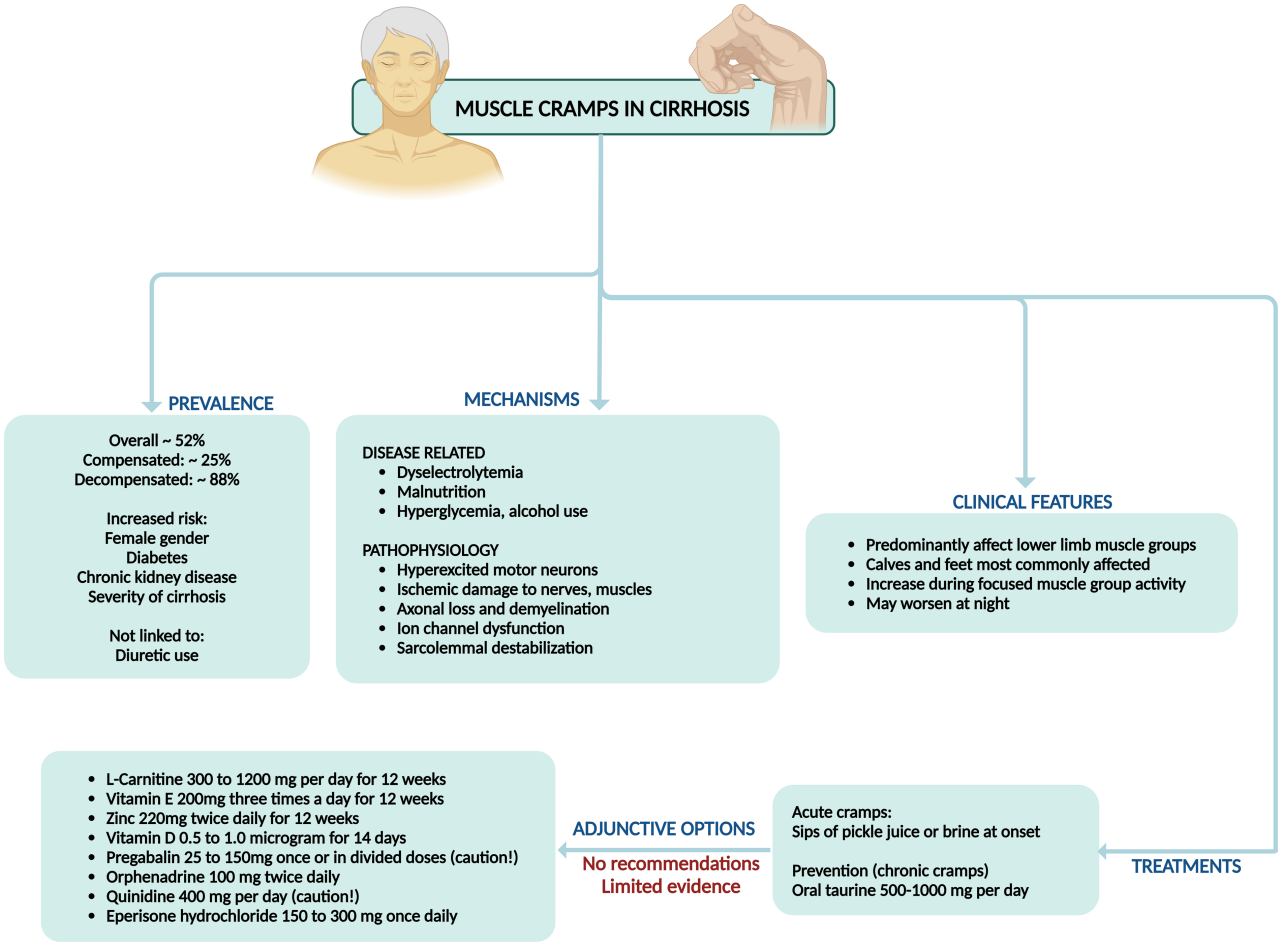
Summary of prevalence, mechanisms, clinical features, and management of muscle cramps in cirrhosis.

Importantly, none of these clinical trials were adequately powered, well-designed, or replicated to confirm the efficacy of these interventions conclusively. Among these, taurine (500–1,000 mg/day) for prevention stands out with relatively better evidence. Additionally, the utility of pickle juice sips at cramp onset has shown promise in ameliorating acute cramps (Figure 5) (30).

## 6 Sleep disorders in cirrhosis

### 6.1 Prevalence, characteristics, and mechanisms

Sleep disturbances worsen quality of life in cirrhosis, demanding vigilant management to uplift health-related quality of life (HRQoL) and clinical outcomes. Up to 80% of cirrhosis patients report poor sleep quality, as evidenced by Pittsburgh Sleep Quality Index (PSQI) scores exceeding 5 (Child–Turcotte–Pugh [CTP] A: 16.9%, CTP B: 26.5%, and CTP C: 56.6%), and around 16 to 52% suffer from excessive daytime sleepiness (EDS), reflected by Epworth Sleepiness Scale (ESS) scores over 10 (CTP A: 17.6%, CTP B: 29.7%, CTP C: 52.8%) (31). Studies have indicated that 47% of cirrhosis patients endure sleep disturbances, while 69% experience both disturbed sleep and depression. Among those with end-stage liver disease, approximately 81% grapple with sleep disorders, often attributed to HE (32). The relationship between sleep quality and cirrhosis severity is complex; some studies reveal a dose–response link, while others do not. Patients with cirrhosis frequently show increased sleep latency, diminished sleep maintenance and efficiency, alongside altered rapid-eye-movement (REM) sleep patterns. Common sleep disorder phenotypes in cirrhosis include insomnia, EDS, obstructive sleep apnea (OSA), and restless leg syndrome (RLS) (31).

Insomnia, defined as difficulty initiating or maintaining sleep, is chiefly diagnosed through patient-reported symptoms. Cirrhosis patients describe it as an inability to fall asleep, fragmented sleep, frequent nocturnal awakenings, and overall poor sleep quality, impairing daytime functioning. Observational studies reveal that insomnia affects 42–65% of cirrhosis patients. Notably, insomnia prevails even in well-compensated cirrhosis stages and is not linked to neuropsychiatric impairment. Research indicates a higher prevalence of insomnia in hepatitis C patients compared to those with hepatitis B or other causes. Insomniac cirrhotics tend to be older and have larger neck circumferences, though no correlation with body mass index has been found (33). Excessive daytime sleepiness is a significant concern among cirrhosis patients with sleep disorders, affecting 21–50% of cases. Studies uncover a robust association between EDS and HE, with 38% of cirrhosis patients experiencing EDS, 89.5% of whom also manifest overt HE. EDS is closely linked to severe cirrhosis and the neuropsychiatric impairments seen in HE (34). Obstructive sleep apnea, caused by upper airway obstruction leading to chronic intermittent hypoxia, maintains a well-documented bidirectional relationship with metabolic syndrome-associated steatotic liver disease (MASLD). It is prevalent in 35–45% of patients with obesity and MASLD due to shared metabolic alterations and comorbidities. Additionally, cirrhosis and viral hepatitis patients show a higher incidence of OSA compared to control groups and patients with severe symptomatic ascites develop sleep disturbances due to secondary OSA (35).

Restless leg syndrome, characterized by an irresistible urge to move the legs during rest accompanied by unpleasant sensations, affects 26.1–55.0% of cirrhosis patients, further disrupting sleep. One study showed that the prevalence was 62% compared to 10% in the general population. The prevalence rates of RLS in cirrhosis vary, with higher rates reported in the United States and Europe compared to Asia (36). Sleep disturbances detrimentally impact HRQoL, correlating with higher mortality and hospitalization rates. Frailty, prevalent in cirrhosis, is linked to increased disease progression and mortality. Poor sleep quality is strongly associated with frailty. Sleep disturbances predict malnutrition and correlate with sarcopenia. Furthermore, sleep disorders in cirrhosis are closely related to HE, with overlapping mechanisms such as disrupted sleep patterns and hyperammonemia (37).

Sleep–wake abnormalities in cirrhotic patients can be attributed to several pathophysiological mechanisms. Delayed sleep onset is linked to decreased melatonin clearance, delayed melatonin peaks, increased melatonin levels during daytime and altered circadian variation of core body temperature. Excessive daytime sleepiness is primarily associated with HE. Short total sleep time, low sleep efficiency, and frequent awakenings are also related to HE, as well as increased interleukin-6 levels, glucose level fluctuations, and low ghrelin values. These disruptions collectively contribute to the compromised sleep health observed in cirrhotic patients (31, 34).

### 6.2 Assessment and treatment

Sleep disturbances in cirrhosis patients can be evaluated by assessing night sleep quality, sleep–wake timing, and daytime sleepiness. The methods for evaluating sleep–wake behavior in these patients is diverse and can be categorized into subjective and objective/semi-quantitative approaches. Subjective methods for evaluating sleep disturbances encompass daily sleep diaries and retrospective questionnaires. Despite the absence of specific protocols for cirrhosis patients, sleep diaries are heralded as the “gold standard” for subjective sleep assessment (31). Carney et al. crafted the “Consensus Sleep Diary,” available in three variations: the “Core Consensus Sleep Diary” with nine essential items, the “Expanded Consensus Sleep Diary for Morning” (CSD-M), which includes early morning awakenings, napping, and substance use, and the “Expanded Consensus Sleep Diary for Evening” (CSD-E) that organizes items for both morning and evening entries (38). The Pittsburgh Sleep Quality Index (PSQI) reigns as the most widely used questionnaire, gaging sleep quality over the past month through 19 items divided into seven components, classifying individuals as “good sleepers” or “poor sleepers” based on a threshold score of 5. The Sleep Timing and Sleep Quality Screening Questionnaire (STSQS) serves as a more concise alternative to the PSQI, providing a rapid assessment that correlates significantly with PSQI outcomes. The Epworth Sleepiness Scale (ESS) measures daytime somnolence across eight scenarios, with scores of 11 or higher denoting excessive daytime sleepiness (EDS). The Basic Nordic Sleep Questionnaire (BNSQ), comprising 27 items, evaluates various sleep complaints and has been employed in studies involving liver transplant recipients and cirrhosis patients. The STOP-Bang questionnaire, with eight items, screens for obstructive sleep apnea (OSA), with scores of 3 or above indicating moderate to severe OSA. Similarly, the Berlin questionnaire (BQ) assesses OSA risk, with a reported 42% risk in cirrhotic patients. Lastly, the International Restless Leg Syndrome Study Group rating scale (IRLSS) appraises the presence and severity of RLS and is a valuable tool specifically in patients with primary biliary cholangitis (31, 33, 36).

Objective methods for sleep disorders assessment in cirrhosis include the polysomnography test which stands as the gold standard for diagnosing sleep disturbances, but its practicality is hindered by high costs and patient compliance challenges. Consequently, alternative assessment methods are often employed. Sleep logs, which track sleep patterns over a minimum of 2 weeks, provide valuable insights. Actigraphy, a portable and cost-effective option, offers a viable alternative to polysomnography, albeit with less accuracy (31–33).

Despite the high prevalence of sleep disturbances among patients with liver cirrhosis, the paucity of routine sleep quality assessments in clinical practice has left a gap in effective management strategies. Current therapeutic options span both pharmacological and behavioral realms. To achieve effective treatment, it is imperative to regularly evaluate nighttime sleep quality and daytime sleepiness using instruments such as the PSQI and the ESS. Lactulose, which lower blood ammonia levels, have demonstrated efficacy in enhancing various sleep parameters even in the absence of overt HE. For example, a three-month course of lactulose therapy significantly improved sleep quality, reduced daytime sleepiness, and increased sleep duration and REM sleep in patients with minimal HE (31, 33). Sedative and hypnotic agents like zolpidem and hydroxyzine have been employed, though their benefits are tempered by potential side effects (31, 35, 39). Modafinil has improved daytime sleepiness in patients with primary biliary cirrhosis and OSA. Melatonin, often deficient in metabolism in cirrhosis patients, has proven effective at a dosage of 3 mg in improving sleep quality and diminishing daytime sleepiness (31, 33, 35). Furthermore, daridorexant, a dual orexin receptor antagonist, has shown promise in extending total sleep duration and reducing daytime sleepiness in cirrhosis patients, though caution is advised for those with advanced cirrhosis (Child C class) (31). Trazodone proves efficacious for insomnia due to its elevated propensity for inducing somnolence compared to second-generation antidepressants, such as mirtazapine (42% versus 25%). It should be contemplated as a primary therapeutic option when insomnia constitutes a significant aspect of somatic symptoms, with mirtazapine serving as a viable alternative. Nonetheless, mirtazapine must be avoided in those with metabolic syndrome and obesity due to strong association with weight gain (approximately 3 kg after 8 weeks use). The undesirable effects associated with tricyclic antidepressants and monoamine oxidase inhibitors constrain their regular application in those with cirrhosis (34).

**Figure 6:**
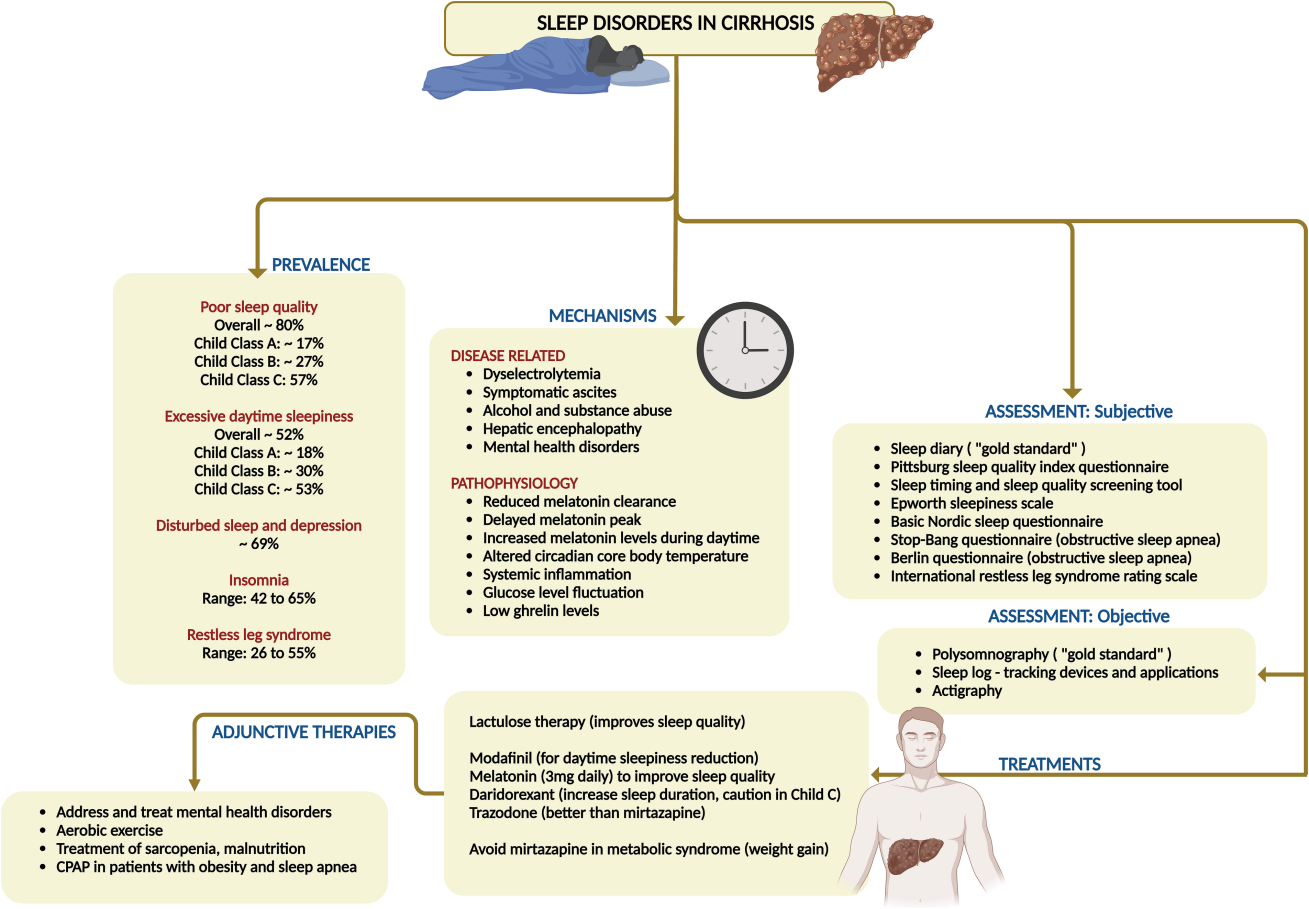
Summary of prevalence, mechanisms, clinical features, assessment, and management of sleep disorders in cirrhosis. CPAP, continuous positive airway pressure.

Continuous positive airway pressure is the treatment of choice for OSA, improving sleepiness and potentially benefiting liver health. Recent findings indicate that rifaximin significantly enhances objective sleep architecture, notably increasing REM sleep, after a 28-day treatment course as evidenced by 24-h polysomnography, although it did not alter subjective perceptions of sleep quality and sleepiness (31, 33). Another intriguing research avenue explored the synergy between the ammonia-lowering agent L-ornithine-L-aspartate (LOLA) and the vigilance-enhancing effects of caffeine. The results revealed that administering LOLA and caffeine effectively controlled the rise in capillary ammonia levels in healthy volunteers. Furthermore, caffeine decreased subjective sleepiness and influenced EEG amplitude across various brain regions, suggesting that this combination deserves deeper exploration in the context of caffeine administration timing in cirrhosis (34, 36). Behavioral therapies advocate for a consistent sleep–wake schedule, morning exposure to bright light, and cognitive behavioral therapy for insomnia, albeit the latter which is seldom available for cirrhosis patients. Additionally, mindfulness-based stress reduction, use of lavender baths and resveratrol, and supportive group therapy even though yielded minimal improvements in sleep quality, have not been conclusively recommended (31, 33, 36). A summary of sleep disorders, their mechanisms and management are shown in Figure 6.

## 7 Mental health disorders in cirrhosis

### 7.1 Prevalence and characteristics

Mental health disorders leading to poor quality of life and emotional distress profoundly impact patients with cirrhosis, leading to increasing severity of symptoms related to liver disease, advanced histological abnormalities, increased mortality rates, and poor adherence to medical treatments (40). Anxiety afflicts 25–45% of chronic liver disease patients, while depression affects a staggering 29–72% (41). The prevalence of depression varies among liver disease diagnoses, with notably higher rates in patients with MASLD and chronic hepatitis C, whereas patients with hepatitis B exhibit depression rates comparable to the general population. Nonetheless, emerging research highlights a stark reality on the other side – the prevalence of liver disease among psychiatric disorders: chronic hepatitis B and C are significantly more common among psychiatric populations than the public. A recent meta-analysis reveals hepatitis B prevalence in severe mental illness ranged from 2.2% in South America to a striking 9.7% in Asia, while in hepatitis C it was from 3.0% in South America to an alarming 17.4% in North America. Cohort studies further underscore this disparity, showing that schizophrenia patients suffer from chronic liver disease at a rate of 7.0%, compared to 6.1% in the general population. In individuals with bipolar disorder, the prevalence of chronic liver disease was as high as 13.9%, which is 2.7 times higher than the general population. Moreover, the current and lifetime prevalence of hepatic illness in bipolar disorder stands at 17 and 21%, respectively (42). Anxiety disorders are rampant among cirrhosis patients, severely diminishing their quality of life. Depression is also rampant in MASLD patients, with 23.6% meeting depression criteria. Another study revealed that patients with MASLD face 3.8 times higher odds of lifetime depression compared to those without liver disease. A recent study showed that nearly 1 in 6 patients with cirrhosis have moderately severe to severe depression and nearly half have moderate–severe anxiety (43). A study investigated the relationship between hepatic diseases and psychiatric symptoms, focusing on the prevalence and impact of mental health disorders in hospitalized cirrhosis patients in the United States from 2002 to 2014 and found that 37% of cirrhosis patients had a mental illness diagnosis, with prevalence rising from 22.6% in 2002 to 54.1% in 2014. Common mental health disorders included a history of mental health issues (49.4%), mood disorders (22.9%), substance use disorders (14.4%), anxiety (9.6%), and schizophrenia (2.4%), with a slight male preponderance (44). Authors from the United States, found that the prevalence of depression in cirrhosis was significantly higher at 23.93% compared to 7.61% in the non-cirrhotic control group. Multivariate analysis revealed that patients with liver cirrhosis had more than twice the risk of developing depression (odds ratio = 2.172), concluding that cirrhosis was an independent risk factor for developing major depressive disorder (45). In another study, mental health diagnoses were prevalent among cirrhosis patients and significantly increased the risk of all-cause mortality: 54% for any mental health diagnosis, 11% for non-alcohol-use disorder (AUD)/substance-use disorder (SUD), and 44% for AUD/SUD. The authors found that regular outpatient mental health visits can mitigate this risk, decreasing all-cause mortality by 21% for AUD/SUD patients, 9% for non-AUD/SUD, and 3% for any mental health diagnosis (46).

There are multiple causal pathways linking mental health disorders and cirrhosis. Inflammation and gut microbiota play crucial roles in both psychological distress and liver disease initiation and progression, while the “sickness behavior theory” explains that peripheral inflammation can lead to behaviors like fatigue, lethargy, impaired concentration, and social withdrawal. If these illness-associated behaviors persist, they can lead to the development of psychological distress, such as clinical depression and anxiety.

### 7.2 Assessment and management

Different scales and instruments, such as the hospital anxiety and depression scale and the generalized anxiety disorder score-7 and the patient health questionnaire-9 help measure anxiety and depressive symptoms in cirrhosis. In contrast, tools like the structured clinical interview for Diagnostic and Statistical Manual of Mental Disorders (DSM) can distinguish clinical diagnoses such as major depressive disorder or generalized anxiety disorder.

Depression ranges from everyday situational depression to major depressive disorder, characterized by profound hopelessness, helplessness, and biological symptoms such as sleep disorders and anhedonia and can be mimicked by symptoms of cirrhosis like HE and delirium, necessitating careful psychiatric differential diagnoses. The key to treatment of mental health disorders such as depression and anxiety is to initiate patients on the lowest doses possible and titrate upward every 2 weeks depending on underlying liver disease severity, patient tolerance, and emergence of adverse events. Nonetheless, the recommended target maintenance dose for most medications in these categories for cirrhosis patients should be half standard dosing. For severe major depressive disorder [identified using a Patient Health Questionnaire (PHQ)-9 score ≥ 20], an integrated approach combining pharmacotherapy and psychotherapy is preferred. In contrast, mild-to-moderate major depressive disorder (PHQ-9 < 20) can be effectively managed with either second-generation antidepressants such as selective serotonin reuptake inhibitors (SSRI) or serotonin and norepinephrine reuptake inhibitors (SNRI) or psychotherapy alone. Second-generation antidepressants are favored for their proven efficacy and safety, with drug selection meticulously tailored to the patient’s specific symptoms, side effects, comorbidities, and personal preferences. The landmark STAR*D study, which included a broadly representative “real-world” patient sample with both medical and psychiatric comorbidities, reported a 37% remission rate with citalopram as the first-line therapy (47). Despite second-generation antidepressants’ low hepatotoxicity risk, caution is warranted when co-administered with non-steroidal anti-inflammatory drugs (due to serotonin transporter inhibition on platelets affecting platelet aggregation) and antiplatelets due to potential bleeding risks. Antidepressants should be commenced at low doses, with maintenance doses for SSRIs/SNRIs set at approximately half the standard dosing for cirrhosis patients to ensure a judicious balance between therapeutic efficacy and adverse effects. Cognitive behavioral therapy and interpersonal psychotherapy stand as the foremost behavioral therapies for depression, backed by robust evidence of their efficacy. The effectiveness of these treatments’ hinges significantly on the patient’s commitment to participate actively. Furthermore, additional therapeutic avenues, including family therapy, problem-solving therapy, relaxation techniques, and exercise programs, can be implemented, customized to the patient’s tolerance and the severity of their condition (42, 48).

Anxiety in patients with cirrhosis poses a significant diagnostic challenge due to overlapping symptoms, necessitating meticulous differential diagnosis to prevent mismanagement. For mild generalized anxiety disorder (GAD; GAD-7score < 10) without functional impairment, regular follow-up may be adequate. However, for more severe cases, first-line treatments include SSRIs/SNRIs and cognitive behavioral therapy, beginning with low doses and adjusting cautiously is mandated. Patients unresponsive to SSRIs/SNRIs may be treated with medications such as buspirone, pregabalin, or hydroxyzine, with careful monitoring for precipitation of HE. If effective, drugs for anxiety disorder must be continued for a maximum of 12 months. If benzodiazepines are deemed necessary for patients with cirrhosis short-acting options like lorazepam, which lack active metabolites, should be preferred to minimize the risk of drug accumulation. Nevertheless, the potential for benzodiazepine dependence restricts their use. Furthermore, cirrhosis patients administered benzodiazepines for three or more days face a heightened risk of first-time HE, likely due to increased cerebral benzodiazepine receptor availability, especially in those with alcohol use disorder. Additionally, medications such as pregabalin, due to its gamma-amino butyric acid (GABA) receptor agonism, and hydroxyzine, due to its anticholinergic effects, can also elevate the risk of HE, necessitating vigilant monitoring of cirrhosis patients on these treatments (42, 48, 49).

**Figure 7:**
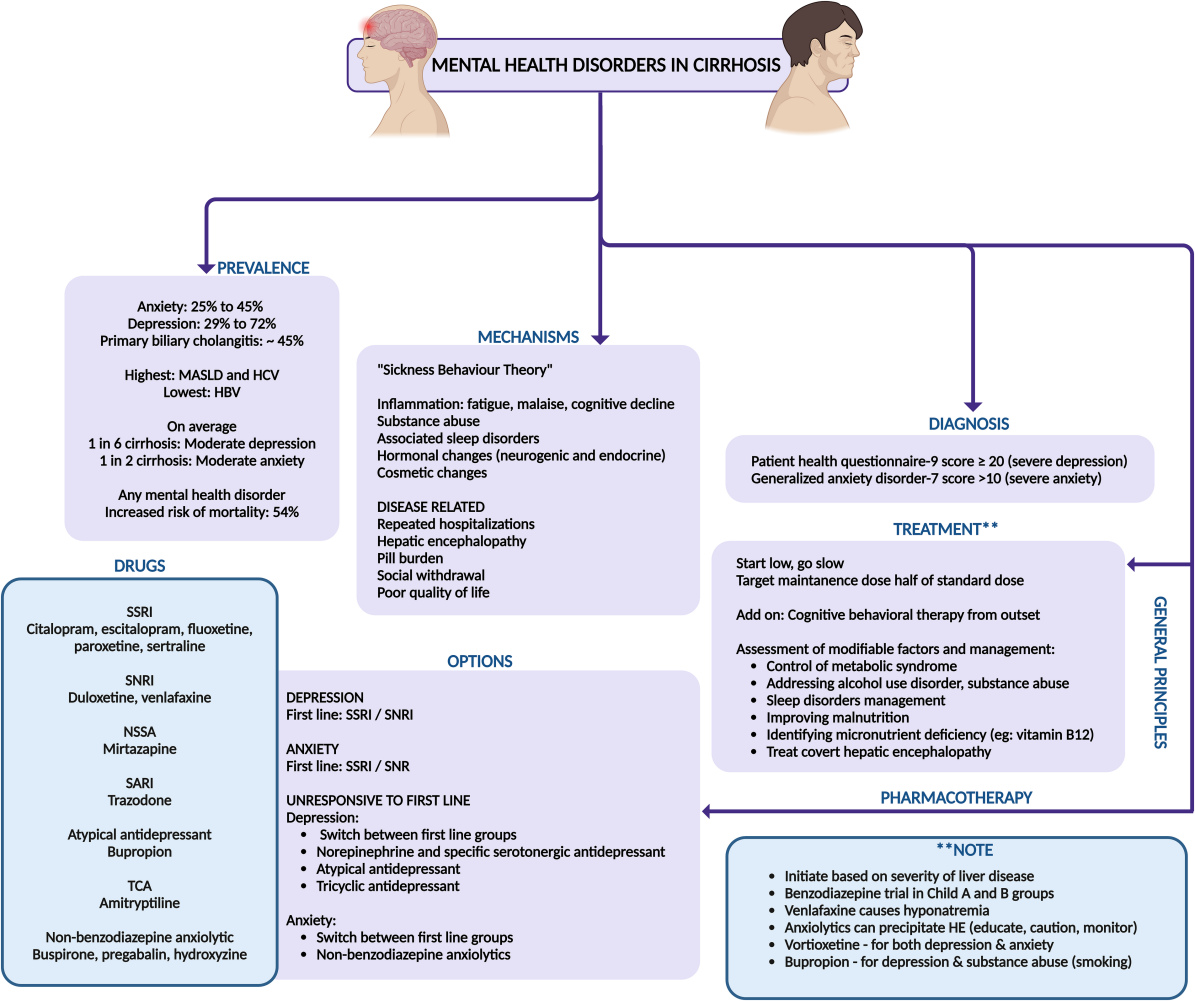
Summary of prevalence, mechanisms, diagnosis, and management of mental health disorders in cirrhosis. SSRI, selective serotonin reuptake inhibitor; SNRI, serotonin–norepinephrine reuptake inhibitor; NSSA, noradrenergic and specific serotonergic antidepressant; SARI, serotonin antagonist and reuptake inhibitor; TCA, tricyclic antidepressants; MASLD, metabolic-syndrome (dysfunction) associated steatotic liver disease; HBV, hepatitis B virus; HCV, hepatitis C virus.

In addition to standard treatment options, less common but increasingly popular medications like vortioxetine and bupropion can be considered for patients with cirrhosis, as they are presumed to be safe for the liver. Vortioxetine, an antidepressant also used for generalized anxiety disorder, falls under the serotonin modulator and stimulator category. It has been associated with a low incidence of minor serum aminotransferase elevations during treatment and has not been linked to clinically apparent acute liver injury. Similarly, bupropion, an aminoketone antidepressant used for depression and smoking cessation, is rarely associated with clinically apparent liver injury (50, 51). Holistic management approaches, including aerobic exercise, mindfulness-based stress reduction, and yoga, are valuable adjunctive therapies, but require further validation for long term compliance and efficacy (42, 48). A summary of management of common mental health disorders in cirrhosis is outlined in Figure 7.

## 8 Gastrointestinal symptoms in cirrhosis

### 8.1 Prevalence and characteristics

Gastrointestinal symptoms worsen quality of life and impact daily routine in the lives of those with cirrhosis with approximately 80% of cirrhotic patients enduring these symptoms. These include abdominal bloating (49.5%), abdominal pain (24%), belching (18.7%), diarrhea (13.3%), and constipation (8%) according to various studies (52). Functional gastrointestinal disorders are common symptom complexes characterized by persistent and recurring gastrointestinal symptoms where no structural or organic pathology is identified and is commonly encountered in cirrhosis population. The intensity of these ailments mirrors the progression of liver disease, lactulose usage, severity and presence of ascites, mental health disorders, sleep abnormalities and psychological distress, as well as diminished serum testosterone levels. Studies have shown that the physical component of QoL assessment revealed a negative correlation with Child-Pugh scores. In cirrhosis, both physical and mental QoL significantly deteriorated with an increasing number of gastrointestinal symptoms. Furthermore, the prevalence of gastrointestinal symptoms was strongly linked to heightened anxiety, depression, and neuroticism scores (53).

The gastrointestinal symptoms are closely associated with a combination of factors, including motility disturbances, visceral hypersensitivity, altered mucosal and immune functions, changes in gut microbiota, and altered central nervous system processing which are highly prevalent in cirrhosis. Patients with cirrhosis frequently contend with premature satiety, curtailing their food intake, resulting in weight loss and related worsening of gastrointestinal symptoms (54). Compared to their healthy peers, cirrhotic patients harbor elevated plasma gastrin levels and a higher prevalence of peptic ulcers, often silent and intertwined with decompensated cirrhosis. *Helicobacter pylori* infections are more prevalent among cirrhotics with peptic ulcers, yet alcohol consumption and portal hypertension also play significant roles. The prevalence of peptic ulcer bleeding and re-bleeding are higher among those with cirrhosis, even though their prognosis during such episodes aligns closely with that of non-cirrhotic individuals (52, 54, 55). Furthermore, gastric sensorimotor function is impaired in those with cirrhosis. The presence of tense ascites disrupts gastric accommodation, while both gastric emptying and small bowel transit are slowed, likely due to imbalances in postprandial glucose, insulin, and ghrelin levels (55). These disturbances are also intertwined with insulin resistance in cirrhosis especially in those with metabolic syndrome. Additionally, delayed gut transit and small bowel manometry anomalies may pave the way for the rise of small bowel bacterial overgrowth and microbiome related changes and associated gastrointestinal dysfunction and related symptoms. Patients with cirrhosis have more obvious gastrointestinal symptoms and higher gastrointestinal hormone levels, which were closely correlated with the progression of liver cirrhosis and the degree of liver function damage (55, 56).

### 8.2 Assessment and management

Gastrointestinal symptoms in cirrhosis can be diagnosed to be secondary to functional causes and should follow the ROME IV criteria for classification and diagnosis. These diagnoses commonly include esophageal disorders (functional heartburn, reflux hypersensitivity, functional dysphagia), gastroduodenal disorders (functional dyspepsia, and nausea and vomiting disorders), bowel disorders (irritable bowel syndrome related constipation, diarrhea or mixed type, functional constipation or diarrhea and bloating/distension), centrally mediated abdominal pain syndrome, functional gallbladder disorder and anorectal disorders (57). It is imperative for the physician to assess the patient with cirrhosis thoroughly to rule out modifiable causes for these symptoms, before targeting treatment of psychosomatic and “functional” aspects of these symptoms. The modifiable causes for amelioration of gastrointestinal symptoms in patients with cirrhosis include: management of ascites, screening for and treating infections, correction of dyselectrolytemia (hyponatremia is a common cause of nausea and bloating in cirrhosis) including hypomagnesemia, drug treatment modification (titration of lactulose or switching to polyethylene glycol for better tolerance and management of HE), reassessing diet and medication history and identifying and stopping offending agents [over the counter use of pain medications causing abdominal pain, medications that promote nausea and vomiting (ursodeoxycholic acid, non-absorbable disaccharides), diarrhea (non-absorbable disaccharides, branched-chain amino acids) or constipation (curbing very high protein diet, over the counter supplements)], control of metabolic syndrome and screening for and managing cardiovascular and pulmonary disorders associated with cirrhosis or etiology of cirrhosis. It is also important for cirrhosis patients to follow a timed, frequent, well-spaced meal plan to improve satiety, target nutritional requirements and prevent gastrointestinal symptoms such as heartburn, reflux, bloating and dyspepsia. The large burden of gastrointestinal symptoms in cirrhosis can be adequately addressed by lifestyle changes and modification in medications. In the event of functional causes for the symptoms, then psychotherapeutic and pharmaceutical treatments to address the risks (sleep abnormalities) and triggers (illness anxiety and other mental health disorders) may be initiated.

Contemporary research illuminates the efficacy of interventions like cognitive behavioral therapy (CBT) and biofeedback-assisted relaxation training (BART) (58). These therapies endeavor to reshape thought patterns and behaviors while bolstering self-efficacy, thereby enhancing overall health outcomes. Cognitive behavioral therapy, anchored in the principle that cognition molds emotions and actions, suggests that transforming thought processes can beneficially influence physical experiences. Negative thought patterns are often linked to adverse physical sensations, such as abdominal pain and discomfort. By retraining these thought processes, CBT can mitigate symptoms and elevate the quality of life (59). Biofeedback-assisted relaxation training therapies concentrate on observing physiological changes associated with thoughts and emotions, teaching patients to visualize the effects of their interventions. This intervention improves mood and somatic responses to anxiety disorders, thus alleviating the psychological and physiological distress that exacerbates gastrointestinal symptoms (60). Nonetheless, the short- and long-term benefits of these modalities in cirrhosis population remain an unmet need.

**Figure 8:**
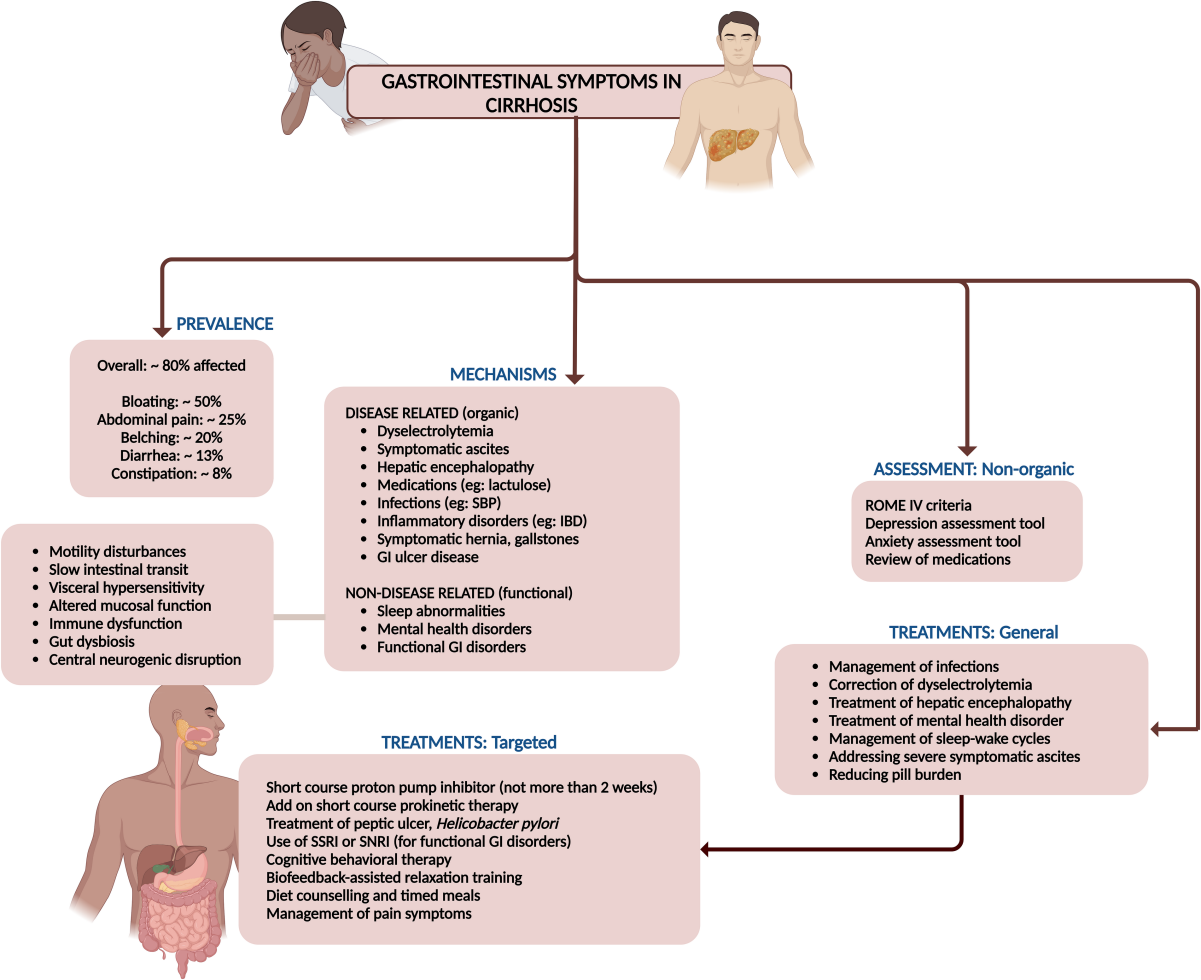
Summary of prevalence, mechanisms, assessment, and management of gastrointestinal symptoms in cirrhosis.

Pharmaceutical treatments, particularly antidepressants, have been meticulously studied for their efficacy in treating functional gastrointestinal symptoms. Tricyclic antidepressants like amitriptyline, selective serotonin reuptake inhibitors (SSRIs), and selective norepinephrine reuptake inhibitors (SNRIs) exhibit significant promise in symptom alleviation. Tricyclic antidepressants and SNRIs are notably effective in managing pain and enhancing quality of life, while SSRIs, though less potent for pain, can reduce anxiety and depression, indirectly soothing gastrointestinal symptoms (58–60). However, as previously discussed, these medications warrant well-designed clinical trials, and, cautious patient-centric use, weighing in factors such as metabolic syndrome, drug interactions, alcohol and substance use disorders, and the severity of liver disease and its complications (Figure 8).

## 9 Sexual dysfunction in cirrhosis

### 9.1 Prevalence, types, risk factors and pathogenesis

Sexual dysfunction (SD) associated with liver diseases and cirrhosis is prevalent in a markedly high proportion of both men and women, yet it is frequently overlooked, underestimated, and ignored. Sexual dysfunction in men can present as low libido and erectile dysfunction (ED), while in women it can manifest as low libido, inability to achieve orgasm, painful intercourse (dyspareunia), as well as various menstrual irregularities such as anovulation, amenorrhea, oligomenorrhea, and dysmenorrhea (61).

For cirrhosis in general, the prevalence of ED was 70.3%, with a positive correlation between ED severity and increasing Child-Pugh (CTP) scores. In chronic viral liver diseases, the prevalence of ED was reported at 60% for individuals under 50 years and 88% for those over 50. Hepatitis B-related liver disease showed a total ED prevalence of 24.6%, with higher rates in HBV-related liver cirrhosis (41.2%). For hepatitis C, the reported prevalence was 30% among patients aged 20–80 years. In alcohol-related liver disease, the prevalence was found to be 61% in men under 56 years. Lastly, chronic liver disease in general, with a mean age of 54.8 years, showed a 50.6% prevalence of ED. Among women cirrhosis on the liver transplant wait list, reportedly, 42% had irregular menstrual cycles, 28% irregular and unpredictable bleeding, and 30% amenorrhea (62).

Men with cirrhosis often exhibit hypogonadism and signs of feminization, including testicular atrophy, low testosterone levels, diminished libido, infertility, reduced secondary sexual hair, and gynecomastia. These symptoms are linked to reduced spermatogenesis and peritubular fibrosis in 50% of cases. In women with cirrhosis, chronic anovulation is prevalent, manifesting as secondary amenorrhea, oligomenorrhea, or irregular episodes of metrorrhagia. Amenorrhea is particularly common in women with both alcohol-related and non-alcohol-related chronic liver disease, and levels of testosterone, oestradiol, prolactin, and luteinizing hormone often differ significantly from those in healthy individuals (63).

The pathogenesis of SD is complex and can result from alterations of the hypothalamic–pituitary–gonadal (HPG) axis due to changes in sex hormone metabolism and encompass a range of physiological and psychological factors. Drug interactions, particularly with nonselective beta-blockers and diuretics like spironolactone, also contribute to SD. Additionally, the direct toxic effects of alcohol and its metabolite acetaldehyde, on the gonads are significant contributors (64). Metabolic syndrome, including diabetes, hypertension, and autonomic neuropathy, plays a role, as does primary testicular dysfunction resulting from hypoestrogenism due to blood shunting in portal hypertension and associated with portosystemic shunt placement. Advanced age, physical changes, sarcopenia, and malnutrition are also implicated causes for SD in cirrhosis among both genders. Furthermore, advanced liver disease itself, characterized by higher liver disease severity scores, high hepatic venous pressure gradient, low serum albumin, and large-volume ascites and presence of neuropsychiatric manifestations of HE significantly impacts sexual function. Lastly, psychological factors like depression, anxiety, and stress, and sleep disorders, which can affect all phases of sexual function, are critical contributors to SD in cirrhosis (65). Additionally, in women with cirrhosis, menopausal symptoms can significantly contribute to sexual dysfunction due to the accompanying vasomotor, psychological, and physical changes. These changes encompass hot flushes, palpitations, insomnia, depression, bladder issues, and vaginal dryness. Consequently, these symptoms adversely impact sexual desire, libido, and arousal—three critical phases of normal female sexual function (66).

### 9.2 Assessment and treatments

For males, sexual dysfunction is measured using the International Index of Erectile Function (IIEF), which encompasses five domains: erectile function (EF), orgasmic function (OF), sexual desire (SD), intercourse satisfaction (IS), and overall satisfaction (OS). The severity of ED is categorized based on IIEF scores, with 5 or lower indicating no attempts at intercourse, 6–10 severe ED, 11–16 moderate ED, 17–25 mild ED, and 26–30 normal erectile function or no ED. Additional assessment methods for males include the Sexual Encounter Profile (SEP) diaries, Global Assessment Question (GAQ), and Quantitative Androgen Deficiency in Aging Males (ADAM) questionnaire (61, 62). For females, sexual dysfunction is assessed using the Female Sexual Function Index (FSFI), covering six domains: desire, arousal, lubrication, orgasm, satisfaction, and pain. An overall FSFI score below 26.55 is indicative of female SD, with scores ranging from 2 to 36. Other methods for assessing female sexual dysfunction include the Golombok Rust Inventory of Sexual Satisfaction, Brief Index of Sexual Functioning for Women, Changes in Sexual Functioning Questionnaire, Derogatis Interview for Sexual Functioning, and the FSFI, validated for use in liver diseases (61, 62).

In general, the evaluation of SD involves the adequate treatment of primary liver diseases, asking leading questions about sexual and psychological issues, and conducting a detailed targeted history along with a careful pelvic examination. Relevant investigations and the use of questionnaires for SD assessment are essential. Treatment of SD focuses on identifying and addressing the primary cause of the dysfunction. This includes providing counseling and psychological support, treating the underlying liver diseases, and offering supportive treatments specifically for SD. Evaluating SD in individuals with cirrhosis requires a thorough assessment, encompassing routine laboratory tests such as complete blood count, coagulation profile and biochemistry (including urea, electrolytes, and serum creatinine), alongside reproductive hormone levels (61–63).

In men, this evaluation entails measuring testosterone, luteinizing hormone (LH), follicle-stimulating hormone (FSH), prolactin, and sex hormone-binding globulin (SHBG). The initial step in diagnosing androgen deficiency is to measure total testosterone from a morning blood sample. If total testosterone is low or borderline and symptoms of reproductive dysfunction are evident, further analysis of free or bioavailable testosterone (including SHBG measurement) and concurrent LH and FSH values is warranted. Reference ranges for reproductive hormones vary with assay platforms, and free testosterone, calculated using Vermeulen’s formula, provides a more precise evaluation of androgen status, particularly in cirrhosis where SHBG production is altered. In cases of hypogonadism, LH and FSH levels help differentiate between primary testicular dysfunction (elevated LH and FSH) and hypothalamic or pituitary insufficiency (low or normal LH and FSH) (61, 62).

For female patients, a detailed menstrual history is imperative, beginning with an evaluation of FSH, LH, prolactin, and thyroid-stimulating hormone. Elevated prolactin necessitates further investigation into hyperprolactinemia, while abnormal thyroid-stimulating hormone levels indicate thyroid dysfunction. Elevated LH and FSH suggest primary ovarian failure, whereas low or normal levels in the absence of regular menses point to potential hypothalamic or pituitary issues, although low-normal LH and FSH can also be observed in polycystic ovarian syndrome and during the normal menstrual cycle. Ideally, FSH and LH should be measured on the third day of the menstrual cycle. For suspected anovulation, measuring serum progesterone within the luteal phase can indicate ovulation if the levels fall within the luteal reference range or exceed 5 ng/mL, though levels below the expected range do not exclude ovulation, particularly if the sample is collected outside day 21, which is common in women with irregular menses (61, 62).

**Figure 9:**
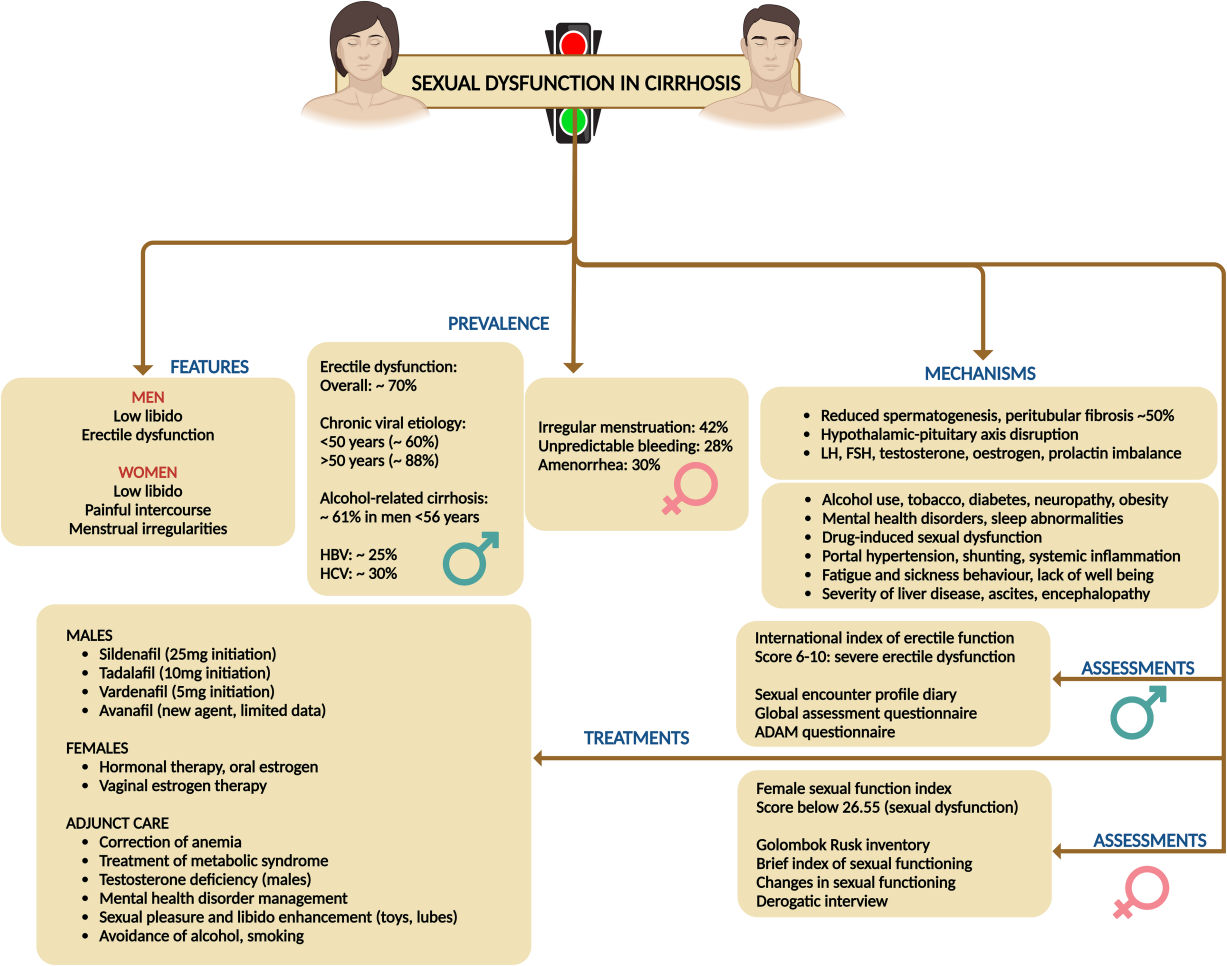
Summary of prevalence, mechanisms, assessment, and treatments related to sexual dysfunction disorders in cirrhosis.

For both genders, the approach emphasizes adequate treatment of primary liver diseases, joint counseling for both partners, environmental modifications to ensure a suitable place and timing for sexual activity, and the avoidance of supplements, including complementary and alternative medicine, and over-the-counter medicines that are not evidence-based. For males, treatment options for ED include the use of phosphodiesterase-5 (PDE5) inhibitors, with caution in Child-Pugh class C patients. Specific medications listed include sildenafil (starting dose 25 mg), tadalafil (dose 10 mg, with longer half-life and better compliance), vardenafil (dose 5 mg, maximum 10 mg), and avanafil, which is a newer agent with limited experience. For females, the treatment strategy covers various aspects of sexual dysfunction. Anovulation and amenorrhea can be managed with oral contraceptive pills or estrogen therapy. Hormone replacement therapy is suggested for menopause. Reduced libido should be treated by identifying and addressing depression and providing psychosocial support. Reduced arousal can be managed with lubricants and inability to orgasm may be aided by clitoral stimulation devices and sex aids. Additionally, lifestyle modifications are advised, such as pelvic floor exercises for women depending on the stage of cirrhosis, and cessation of smoking and alcohol consumption for both genders. To address anemia-related performance issues in sexual activity, blood transfusions or intravenous iron therapy may be necessary. Forced sexual acts may increase the risk of bleeding and must be cautioned against. Strategies to enhance arousal include couples counseling, sex education with videos and literature, education on sexual positions, creating a conducive environment for sexual pleasure, and extending foreplay time. Cognitive behavioral therapy can also help by removing sexual inhibitions and enhancing sexual involvement by positively targeting interpersonal relationships (Figure 9) (61–63, 65).

## 10 Pain and peripheral neurological symptoms

### 10.1 Prevalence and clinical characteristics

Recurrent or chronic pain is a prevalent symptom among individuals with cirrhosis, frequently necessitating the prescription of analgesic medications. A systematic review encompassing five studies revealed that the prevalence of pain in patients with end-stage liver disease varied between 30 and 79% (3). Moreover, a database study from the Veterans Health Administration, indicated a temporal increase in the annual percentage of patients receiving opioid prescriptions, rising from 36% in 2005 to 47% in 2014 and mental health disorders and hepatic decompensation were independently associated with long-term opioid prescriptions (66). Reporting of pain in at least one region was notable in 75.6% patients in a study and chronic pain and wide spreading pain were associated with mood and cognitive disturbance, fatigue, sleep difficulty, and physical and social functioning in patients with cirrhosis (67). Chronic pain is reported by 40–79% of patients with cirrhosis and a pertinent influence on poor functional status and quality of life (68).

There are different phenotypes of pain symptom in cirrhosis which has their own pathophysiology and treatment options and responses. Pain is classified acute (≤3 months) or chronic types (>3 months) and into nociceptive, neuropathic, and nociplastic categories, each demarcated by distinctive mechanisms, clinical manifestations, diagnostic protocols, and therapeutic interventions.

Nociplastic pain, stemming from altered nociception without evident tissue damage, manifests as pervasive, fluctuating pain with accompanying symptoms of sleep disturbances, psychological distress, cognitive impairments, and fatigue. Fibromyalgia serves as the quintessential example, also observed in patients with early or advanced cirrhosis. Patients typically report fluctuating, widespread dull or aching pain, which may also include neuropathic characteristics such as burning or tingling. They often experience concurrent issues like sleep disturbances, psychological distress, memory problems, fatigue, and heightened sensitivity to sensory stimuli like light or unpleasant smells. A patient’s history may reveal a prolonged struggle with pain and associated symptoms that have been resistant to conventional analgesics or treatments targeting peripheral pain, such as injections. Additional indicators of nociplastic pain include extensive healthcare utilization, high levels of pain-related distress, and a family history of chronic pain or psychological disorders. Psychiatric conditions, especially mood disorders, frequently coexist with nociplastic pain, suggesting a bidirectional relationship due to shared risk factors like trauma and common disease mechanisms involving neurotransmitters. The diagnosis is primarily clinical, although questionnaires can help evaluate pain location and severity. The 2016 Fibromyalgia Survey Criteria, a self-report tool, assesses pain distribution via the widespread pain index and symptom severity via the symptom severity scale, with the resulting Fibromyalgia Severity Score serving as a measure of central sensitization (69–72).

Nociceptive pain, arising from actual or imminent tissue injury, encompasses well-defined somatic pain or diffuse visceral discomfort accompanied by autonomic symptoms such as nausea and diaphoresis. This type of pain is diagnosed via meticulous physical examination and imaging studies. Examples include trauma and surgical interventions, and specifically in cirrhosis, conditions such as fractures, severe ascites, discomfort of splenomegaly, muscle cramps, musculoskeletal disorders-related pain, and tender gynecomastia induced by aldosterone antagonists. Somatic nociceptive pain is typically well-localized, reproducible, and proportional to the causative injury, while visceral nociceptive pain tends to be diffuse, with referred pain to superficial regions and often accompanied by autonomic symptoms such as sweating, changes in heart rate, and nausea. Pain descriptors with high specificity for nociceptive pain include terms like “heavy, stinging, lacerating, and suffocating.” Although diagnosing nociceptive pain can be complex due to the potential presence of mixed pain components—nociceptive, neuropathic, and nociplastic—a definitive physical examination or diagnostic testing, such as imaging, can often identify a treatable source of pain (73–75).

Neuropathic pain, precipitated by lesions or pathologies of the somatosensory system, is characterized by aberrant sensations following neuroanatomical pathways, diagnosed through comprehensive physical evaluations, confirmatory testing, and instruments like the Neuropathic Pain Questionnaire and painDETECT screening tool. This pain, characterized by originating from regions with abnormal sensation, may present as pain in the absence of stimuli, pain from normally nonpainful stimuli (allodynia), or disproportionate pain in response to harmful stimuli (hyperalgesia). The pain can range from tingling and numbness to sharp, stabbing sensations and may exhibit temperature variations such as burning or cold. Neuropathic pain typically adheres to a neuroanatomical distribution. Should a patient display neuropathic pain, particularly allodynia in a glove and stocking distribution, alongside dysautonomia symptoms like orthostatic hypotension or micturition disorders, small fiber neuropathy should be suspected (76–79).

Cirrhosis presents formidable challenges in the realm of pain management due to the absence of a consensus on treatment protocols and the constrained pharmacologic arsenal. Recommendations advocate for a restrained use of acetaminophen (≤2 g per day) and avoidance of NSAIDs and opioids. The peril associated with any pharmacologic intervention in cirrhotic patients is considerable, given the propensity of NSAIDs to exacerbate ascites, renal impairment, and gastrointestinal hemorrhage. Even a brief NSAID regimen can significantly impair renal function, diminish the effectiveness of diuretics like furosemide, and disrupt hemostasis through inhibited platelet aggregation and thromboxane B2 synthesis. Opioids are frequently co-prescribed with benzodiazepines, compounding the risk of adverse outcomes, including falls and potential overdose fatalities. Thus, a judicious selection of pain management strategies is crucial to ameliorate pain control while mitigating severe complications (67, 68, 80).

### 10.2 Mechanisms and epidemiology

Nociplastic pain stems from central sensitization, involving abnormal pain processing in the peripheral and central nervous systems, leading to increased sensitivity and reduced inhibition. Quantitative sensory testing reveals heightened temporal summation and diminished conditioned pain modulation in chronic pain conditions, indicating supraspinal mechanisms. Imaging studies show structural and functional CNS alterations, highlighting CNS dysfunction as a key pain driver. Recognizing nociplastic pain is vital in cirrhosis, where analgesic decisions are complex. Research indicates that 27% of cirrhosis patients exhibit fibromyalgia symptoms, a quintessential nociplastic pain condition, often accompanied by mood and sleep disturbances indicative of centralized pain states (67, 68). Cognitive impairments are also widespread, ranging from subtle executive function deficits to severe disorientation, with minimal hepatic encephalopathy present in up to 80% of cirrhosis patients. These findings suggest a substantial degree of central sensitization in cirrhosis, highlighting nociplastic pain as a crucial component of chronic pain in these patients (68, 81).

Nociceptive pain, the most comprehended form of pain, emerges from actual or potential tissue damage due to nociceptor activation within a normal sensory nervous system. While nociceptive pain can contribute to chronic pain, it predominantly presents as acute pain, subsiding once the injury heals and inflammation diminishes. In cirrhotic patients, nociceptive pain arises from various sources such as pathological fractures, ascites, splenomegaly, muscle cramps, musculoskeletal diseases like avascular necrosis and septic arthritis, and mastalgia (67, 68, 82).

Neuropathic pain, stemming from lesions or maladies afflicting the somatosensory nervous system, precipitates aberrant sensory processing within the brain and spinal cord. This encompasses a myriad of conditions, including diabetic neuropathy, painful polyneuropathy, painful radiculopathy, trigeminal neuralgia, and small fiber neuropathy. Peripheral neuropathy, frequently associated with neuropathic pain symptoms, is notably prevalent in cirrhosis, irrespective of diabetes or alcohol abuse. Although the precise prevalence of painful peripheral neuropathy in cirrhosis remains elusive, research has revealed that 15% of cirrhotic patients manifest neuropathic symptoms such as numbness and paraesthesia (67, 68).

### 10.3 Treatment and adjunctive management

General principles include addressing comorbid symptoms (such as sleep, mood, memory, fatigue, and psychiatric disorders), emphasizing self-management tools, and employing multimodal therapies that combine several non-pharmacologic strategies. Self-management domains cover emotions, cognitions, behaviors, sleep, and environment, with strategies like mindfulness, pleasant activities, social support, and behavioral sleep techniques (67, 68, 83).

For managing nociceptive pain, pharmacologic treatments include the administration of acetaminophen at a dosage of 500 mg every 6 h, with a maximum limit of 2 g per day. Topical NSAIDs like diclofenac gel are recommended (due to very low systemic absorption). Opioids such as oxycodone, dosed at 2.5 mg as needed, and hydromorphone at 1 mg every 6 h as needed should only be used for acute pain that is severe or unresponsive to initial therapies not for more than 3 days. It is crucial to ensure an effective bowel regimen to counteract opioid-induced constipation. Interventional treatments for nociceptive pain may involve surgical interventions to address the peripheral pain source, as well as injections, nerve blocks, neurostimulation, and intra-articular injections (68, 80, 84).

**Figure 10:**
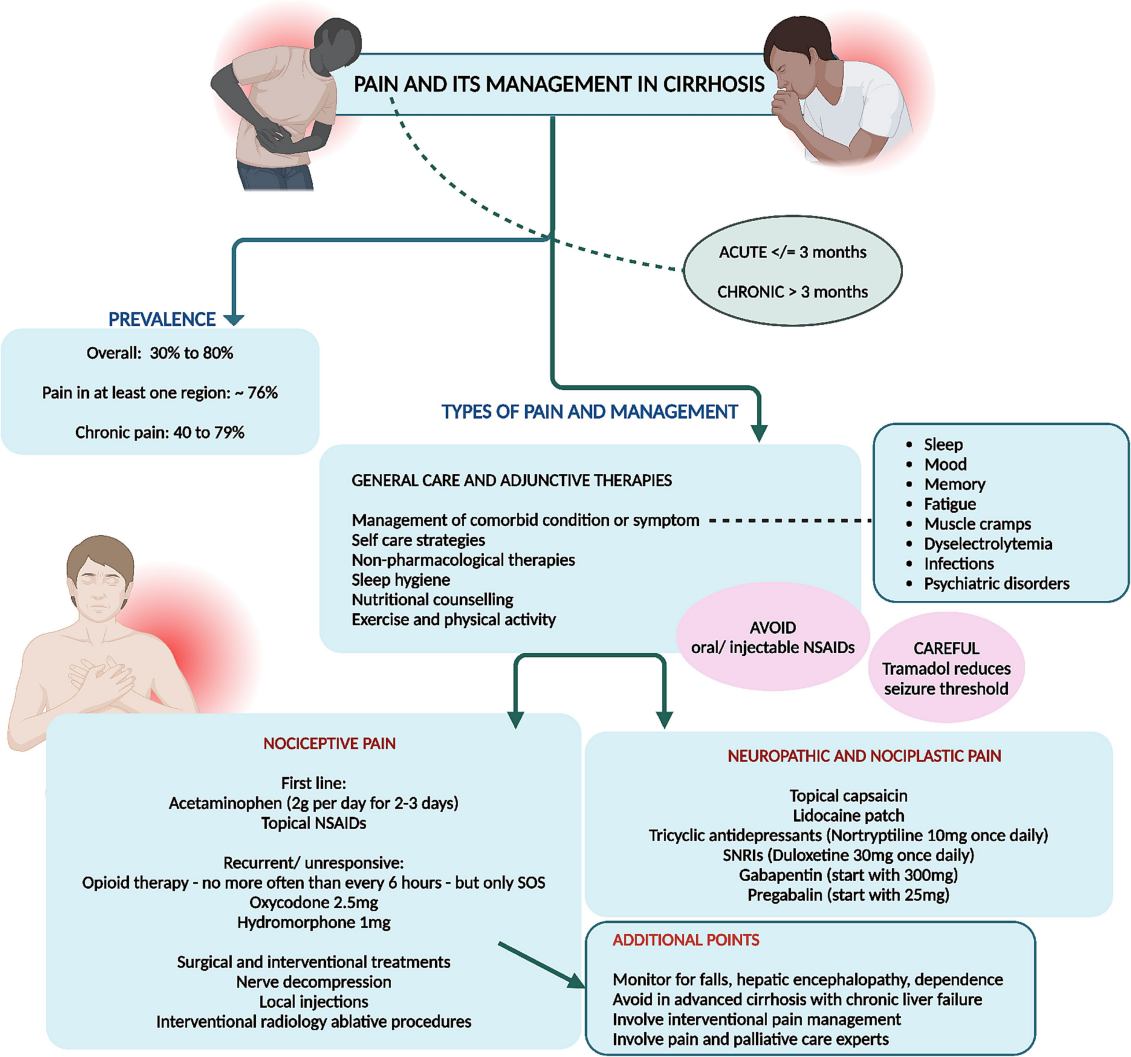
Summary of various types of pain, its assessment and management in cirrhosis. NSAIDs, non-steroidal anti-inflammatory drugs.

In the case of neuropathic or nociplastic pain, pharmacologic options include the use of lidocaine patches and topical capsaicin. Tricyclic antidepressants, such as cyclobenzaprine are dosed at 5–20 mg or nortriptyline, initiated at a dose of 10 mg at bedtime, while SNRIs like duloxetine and venlafaxine are started at 30 mg and 37.5 mg daily, respectively, titrated to a maximum of 50% of that used in non-cirrhotic population. Tramadol is considered to have a more favorable side effect profile compared to other opioids. However, data supporting this are scarce in cases of cirrhosis. Additionally, tramadol can lower the seizure threshold and poses a risk of serotonin syndrome when taken with other medications (83). Gabapentinoids, including gabapentin starting at 300 mg daily and pregabalin at 50 mg twice daily, are also utilized. It is imperative to monitor patients for sedation and fall risks associated with these medications (68, 79, 85). Treatment of nociplastic pain must also include adjunct cognitive behavioral therapies or physical therapy alongside an approved therapist. Specific diet, alternative manual therapies such as acupressure and acupuncture that are based on pseudoscientific principles are not studied via well-designed trials or found beneficial for pain management in cirrhosis population are not recommended. These judicious approaches categorizing pain types aims to tailor management to the individual needs of cirrhotic patients, ensuring a comprehensive and multifaceted care plan that addresses both the physical and psychological aspects of chronic pain. A summary of pain management in cirrhosis is shown in Figure 10.

## 11 Hair loss and skin changes

### 11.1 Prevalence and clinical characteristics

Even though the actuarial prevalence of hair loss and its types, and major skin changes and its types related to symptom-burden in cirrhosis is not well documented or studied, generalized complaints of hair loss and skin changes are routine discussion inside the liver outpatient. Hair loss and significant skin changes increase consciousness and affect mood, emotions, and mental health of the patient with cirrhosis.

Alopecia refers to the absence or loss of hair in areas where hair is typically present and is a common symptom and sign in cirrhosis. This condition can be either localized or widespread, and may be temporary or permanent. It affects both men and women across all age groups. Alopecia is recognized as a symptom arising from a variety of causes and is broadly categorized into nonscarring (the most common type and most common notable in cirrhosis) and scarring (cicatricial) forms. The impact of alopecia can cause considerable emotional distress, significantly diminishing the quality of life for those affected (86, 87).

Hair loss, specifically the loss of pubic hair or beard, is linked to cirrhosis and is associated with elevated estrogen levels. Gynecomastia, characterized by breast enlargement, occurs in 44% of cirrhosis cases, also due to elevated estrogen levels, and is related to conditions like extreme obesity, hypogonadism, and various adrenal and testicular diseases (88, 89). Cirrhosis also leads to pigmentation changes, with 46.9% of patients experiencing blotchy or diffuse muddy gray hyperpigmentation. Leg ulcers in cirrhosis patients result from necrotic tissue due to hypoxia and venous reflux obstruction, with similar occurrences in chronic venous insufficiency and diabetes (89, 90). Lastly, coagulation defects in cirrhosis, manifesting as petechiae, ecchymoses, or mucosal bleeding, are due to thrombocytopenia and other hemostasis abnormalities, like those seen in hematologic disorders and anticoagulant therapy for thrombotic diseases. Dermatological vascular manifestations in cirrhosis include palmar erythema, characterized by blanchable redness in the thenar eminence and fingertips, is seen in 23% of cirrhosis cases due to changes in peripheral hemodynamics and increased estrogen levels, with similar conditions found in rheumatoid arthritis and diabetes. Spider angioma, involving central arterioles surrounded by radiating capillaries, occurs in 33% of alcohol-associated cirrhosis cases and is also linked to hepatopulmonary syndrome, arising from increased blood flow to the surface capillaries. Arteriovenous haemangiomas, presenting as bluish erythematous papules on the neck and chest, are associated with elevated estrogen levels in cirrhosis (91, 92).

### 11.2 Broad principles of evaluation and management

Female cirrhosis patients are most conscious about hair loss. The most common type of hair loss noted in cirrhosis population is telogen effluvium, a noninflammatory alopecia, impacts the entire scalp and can be triggered by stress, illness, pregnancy, malnutrition, infections, endocrine disorders, surgeries, and certain medications. Hair loss usually occurs 3 months post-trigger and resolves within 6 months. A positive hair-pull test conducted at various scalp locations indicates active shedding linked to telogen effluvium. Light microscopy examination of hair shafts can confirm these as telogen hairs. Dermoscopy can help differentiate chronic telogen effluvium, characterized by hair loss lasting over 6 months, from female-pattern hair loss, which shows greater variability in hair diameter. Additionally, the wash test or modified wash test can be useful in evaluating the severity of the condition (89, 93, 94). Telogen effluvium is reversible when an identifiable trigger is controlled. The hair shedding takes 3–6 months to cease and cosmetically significant regrowth may take 12–18 months. Additionally, correcting hypo-functioning thyroid, treating iron and zinc deficiency (and other documented macro and micronutrient deficiencies) also help ameliorate symptoms (94, 95).

Tamoxifen, an estrogen blocker, is effective in treating recent-onset and tender gynecomastia (after stopping or switching spironolactone) when administered at doses of 10–20 mg twice daily. Generally, up to 80% of patients experience partial to complete resolution of symptoms. It is generally used for 3 months before considering a surgical referral, the latter performed for cosmetic reasons and only in stable cirrhosis patients after weighing risk to benefit ratio (96, 97).

Symptomatic skin changes that are part of patient-related complaints mostly include localized or generalized hyperpigmentation and darkening over face or body parts. There are no recommended treatment options in this scenario unless an identifiable cause for the skin condition is present. Skin lightening creams and intravenous or oral glutathione do not offer reasonable clinical benefit over placebo effect, are not associated with long term improvements, harbor potential adverse events (heavy metal toxicity and renal injury) or lack safety data (intravenous glutathione) and hence, not recommended (98–100).

## 12 Conclusion

This comprehensive review delves into the diverse array of symptoms and their management in patients with cirrhosis, underscoring the profound impact these symptoms have on quality of life. Commonly encountered symptoms include pain, muscle cramps, sleep disturbances, psychological symptoms, and gastrointestinal issues. Pain, prevalent in 30–79% of patients, often necessitates analgesics, with chronic pain linked to poor functional status. Muscle cramps, occurring in up to 68% of cirrhosis patients, are treated with medications like zinc, taurine, and branched-chain amino acids, although robust evidence for these treatments is lacking. Sleep disturbances affect up to 80% of cirrhosis patients, with insomnia and excessive daytime sleepiness being particularly prevalent. Management includes lactulose, melatonin, and behavioral therapies and mindfulness. Gastrointestinal symptoms are widespread, affecting 80% of patients, and include abdominal pain, bloating, and diarrhea. These symptoms are closely tied to the severity of liver disease and significantly diminish quality of life. Management strategies emphasize lifestyle modifications, dietary adjustments, and psychological support. Mental health disorders, including depression and anxiety, are common, exacerbating the burden of cirrhosis. Treatment involves a combination of pharmacotherapy and psychotherapy, tailored to individual patient needs. Sexual dysfunction, affecting both men and women, is often overlooked but significantly impacts quality of life. Management includes addressing underlying causes, lifestyle changes, and counseling. Pruritus, a common and distressing symptom, is managed with moisturizers, antihistamines, and other medications like cholestyramine, though many treatments have limited efficacy. Hair loss and skin changes also contribute to the psychological burden of cirrhosis, necessitating a holistic approach to patient care. Overall, this review emphasize the need for comprehensive, multidisciplinary management strategies to address the complex symptomatology in cirrhosis, aiming to improve patient outcomes and quality of life.

## References

[1] Lee MJ. A review of liver fibrosis and cirrhosis regression. J Pathol Transl Med. (2023) 57:189–95. doi: 10.4132/jptm.2023.05.24, PMID: 37461143 PMC10369136

[2] Abraldes JG Caraceni P Ghabril M Garcia-Tsao G. Update in the treatment of the complications of cirrhosis. Clin Gastroenterol Hepatol. (2023) 21:2100–9. doi: 10.1016/j.cgh.2023.03.019, PMID: 36972759 PMC11097249

[3] Peng JK Hepgul N Higginson IJ Gao W. Symptom prevalence and quality of life of patients with end-stage liver disease: a systematic review and meta-analysis. Palliat Med. (2019) 33:24–36. doi: 10.1177/0269216318807051, PMID: 30345878 PMC6291907

[4] Tapper EB Parikh ND. Diagnosis and Management of Cirrhosis and its Complications: a review. JAMA. (2023) 329:1589–602. doi: 10.1001/jama.2023.5997, PMID: 37159031 PMC10843851

[5] Tanaka Y Ikeda K Kaneko Y Ishiguro N Takeuchi T. Why does malaise/fatigue occur? Underlying mechanisms and potential relevance to treatments in rheumatoid arthritis. Expert Rev Clin Immunol. (2024) 20:485–99. doi: 10.1080/1744666X.2024.2306220, PMID: 38224064

[6] Finsterer J Mahjoub SZ. Fatigue in healthy and diseased individuals. Am J Hosp Palliat Care. (2014) 31:562–75. doi: 10.1177/1049909113494748 23892338

[7] Mostafa AM Hafez SM Abdullah NM Fouad Y. Fatigue, depression, and sleep disorders are more prevalent in patients with metabolic-associated fatty liver diseases. Eur J Gastroenterol Hepatol. (2024) 36:665–73. doi: 10.1097/MEG.0000000000002752, PMID: 38477854

[8] Kaplan A Rosenblatt R. Symptom Management in Patients with cirrhosis: a practical guide. Curr Treat Options Gastroenterol. (2022) 20:144–59. doi: 10.1007/s11938-022-00377-y, PMID: 35313484 PMC8928010

[9] Bhandari K Kapoor D. Fatigue in cirrhosis. J Clin Exp Hepatol. (2022) 12:617–24. doi: 10.1016/j.jceh.2021.08.028, PMID: 35535102 PMC9077229

[10] Younossi ZM Kremer AE Swain MG Jones D Bowlus C Trauner M. Assessment of fatigue and its impact in chronic liver disease. J Hepatol. (2024) 81:726–42. doi: 10.1016/j.jhep.2024.04.008 38670320

[11] Fabi A Bhargava R Fatigoni S Guglielmo M Horneber M Roila F. ESMO guidelines committee. Cancer-related fatigue: ESMO clinical practice guidelines for diagnosis and treatment. Ann Oncol. (2020) 31:713–23. doi: 10.1016/j.annonc.2020.02.016, PMID: 32173483

[12] Cheung K Lee SS Raman M. Prevalence, and mechanisms of malnutrition in patients with advanced liver disease, and nutrition management strategies. Clin Gastroenterol Hepatol. (2012) 10:117–25. doi: 10.1016/j.cgh.2011.08.016 21893127

[13] Stirnimann J Stirnimann G. Nutritional challenges in patients with advanced liver cirrhosis. J Clin Med. (2019) 8:1926. doi: 10.3390/jcm8111926, PMID: 31717529 PMC6912781

[14] Ishizu Y Ishigami M Honda T Imai N Ito T Yamamoto K. Decreased appetite is associated with the presence of sarcopenia in patients with cirrhosis. Nutrition. (2022) 103-104:111807. doi: 10.1016/j.nut.2022.111807, PMID: 36029694

[15] Deems RO Friedman MI Friedman LS Munoz SJ Maddrey WC. Chemosensory function, food preferences and appetite in human liver disease. Appetite. (1993) 20:209–16. doi: 10.1006/appe.1993.1021, PMID: 8373143

[16] Marchesini G Bianchi G Lucidi P Villanova N Zoli M De Feo P. Plasma ghrelin concentrations, food intake, and anorexia in liver failure. J Clin Endocrinol Metab. (2004) 89:2136–41. doi: 10.1210/jc.2003-031771, PMID: 15126531

[17] WangTShenJ. Usefulness of simplified nutritional appetite questionnaire (SNAQ) in appetite assessment in elder patients with liver cirrhosis. J Nutr Health Aging. (2018) 22:911–5. doi: 10.1007/s12603-018-1086-5, PMID: 30272092

[18] Chapman B Sinclair M Gow PJ Testro AG. Malnutrition in cirrhosis: more food for thought. World J Hepatol. (2020) 12:883–96. doi: 10.4254/wjh.v12.i11.883, PMID: 33312416 PMC7701970

[19] Fisher M Zimmerman J Bucher C Yadlosky L. ARFID at 10 years: a review of medical, nutritional and psychological evaluation and management. Curr Gastroenterol Rep. (2023) 25:421–9. doi: 10.1007/s11894-023-00900-w, PMID: 37755631

[20] Selim R Ahn J. Pruritus in chronic liver disease. Clin Liver Dis. (2023) 27:47–55. doi: 10.1016/j.cld.2022.08.011 36400466

[21] Oeda S Takahashi H Yoshida H Ogawa Y Imajo K Yoneda M. Prevalence of pruritus in patients with chronic liver disease: a multicenter study. Hepatol Res. (2018) 48:E252–62. doi: 10.1111/hepr.12978, PMID: 28877392

[22] Roh YS Choi J Sutaria N Kwatra SG. Itch: epidemiology, clinical presentation, and diagnostic workup. J Am Acad Dermatol. (2022) 86:1–14. doi: 10.1016/j.jaad.2021.07.076, PMID: 34428534 PMC8678917

[23] Bhalerao A Mannu GS. Management of pruritus in chronic liver disease. Dermatol Res Pract. (2015) 2015:295891. doi: 10.1155/2015/295891, PMID: 25861254 PMC4377431

[24] Yoshikawa S Asano T Morino M Matsumoto K Kashima H Koito Y. Pruritus is common in patients with chronic liver disease and is improved by nalfurafine hydrochloride. Sci Rep. (2021) 11:3015. doi: 10.1038/s41598-021-82566-w, PMID: 33542298 PMC7862656

[25] Nakanishi H Kurosaki M Izumi N. Mechanisms and treatment for muscle cramps in liver cirrhosis In: Yoshiji H Kaji K, editors. The evolving landscape of liver cirrhosis management. Singapore: Springer (2019)

[26] Mehta SS Fallon MB. Muscle cramps in liver disease. Clin Gastroenterol Hepatol. (2013) 11:1385–1391; quiz e80. doi: 10.1016/j.cgh.2013.03.017 23542334 PMC10963048

[27] Murata A Hyogo H Nonaka M Sumioka A Suehiro Y Furudoi A. Overlooked muscle cramps in patients with chronic liver disease: in relation to the prevalence of muscle cramps. Eur J Gastroenterol Hepatol. (2019) 31:375–81. doi: 10.1097/MEG.0000000000001294, PMID: 30362994

[28] Kalia S Nath P Pathak M Anand AC. Treatment of muscle cramps in patients with cirrhosis of liver: a systematic review. J Clin Exp Hepatol. (2022) 12:980–92. doi: 10.1016/j.jceh.2021.10.147, PMID: 35677500 PMC9168737

[29] Gonzalez JJ Tapper EB. Muscle cramps in cirrhosis. Clin Liver Dis (Hoboken). (2024) 23:e0116. doi: 10.1097/CLD.0000000000000116, PMID: 38283307 PMC10810579

[30] Tapper EB Salim N Baki J Zhao Z Sundaram V Patwardhan V. Pickle juice intervention for cirrhotic cramps reduction: the PICCLES randomized controlled trial. Am J Gastroenterol. (2022) 117:895–901. doi: 10.14309/ajg.0000000000001781, PMID: 35416793 PMC11214544

[31] Hui Y Chen X Sun C. Sleep disturbances in patients with cirrhosis: pursuing just right. Port Hypertens Cirrhos. (2023) 2:181–91. doi: 10.1002/poh2.61

[32] Zhao X Wong P. Managing sleep disturbances in cirrhosis. Scientifica (Cairo). (2016) 2016:6576812–5. doi: 10.1155/2016/6576812, PMID: 27242950 PMC4868900

[33] Plotogea OM Ilie M Bungau S Chiotoroiu AL Stanescu AMA Diaconu CC. Comprehensive overview of sleep disorders in patients with chronic liver disease. Brain Sci. (2021) 11:142. doi: 10.3390/brainsci11020142, PMID: 33499194 PMC7911845

[34] Shah NM Malhotra AM Kaltsakas G. Sleep disorder in patients with chronic liver disease: a narrative review. J Thorac Dis. (2020) 12:S248–60. doi: 10.21037/jtd-cus-2020-012, PMID: 33214928 PMC7642630

[35] Formentin C Garrido M Montagnese S. Assessment and Management of Sleep Disturbance in cirrhosis. Curr Hepatol Rep. (2018) 17:52–69. doi: 10.1007/s11901-018-0390-1, PMID: 29876197 PMC5966474

[36] Bruyneel M Sersté T. Sleep disturbances in patients with liver cirrhosis: prevalence, impact, and management challenges. Nat Sci Sleep. (2018) 10:369–75. doi: 10.2147/NSS.S186665, PMID: 30464664 PMC6220431

[37] Yoshiji H Nagoshi S Akahane T Asaoka Y Ueno Y Ogawa K. Evidence-based clinical practice guidelines for liver cirrhosis 2020. J Gastroenterol. (2021) 56:593–619. doi: 10.1007/s00535-021-01788-x, PMID: 34231046 PMC8280040

[38] Carney CE Buysse DJ Ancoli-Israel S Edinger JD Krystal AD Lichstein KL. The consensus sleep diary: standardizing prospective sleep self-monitoring. Sleep. (2012) 35:287–302. doi: 10.5665/sleep.1642, PMID: 22294820 PMC3250369

[39] Rogal SS Hansen L Patel A Ufere NN Verma M Woodrell CD. AASLD practice guidance: palliative care and symptom-based management in decompensated cirrhosis. Hepatology. (2022) 76:819–53. doi: 10.1002/hep.32378 35103995 PMC9942270

[40] Nash R Golden E Dew MA DiMartini AF. Mental health in chronic and end-stage liver disease In: Sher Y Maldonado J, editors. Psychosocial Care of end-Stage Organ Disease and Transplant Patients. Cham: Springer (2019)

[41] Crone CC Gabriel GM DiMartini A. An overview of psychiatric issues in liver disease for the consultation-liaison psychiatrist. Psychosomatics. (2006) 47:188–205. doi: 10.1176/appi.psy.47.3.188, PMID: 16684936

[42] Holmes R Patel A Desai AP. Psychiatric disorders and their treatment: impact of outcomes in patients with chronic liver disease. Clin Liver Dis (Hoboken). (2022) 20:32–7. doi: 10.1002/cld.1204, PMID: 36033426 PMC9405520

[43] Hernaez R Kramer JR Khan A Phillips J McCallister K Chaffin K. Depression and anxiety are common among patients with cirrhosis. Clin Gastroenterol Hepatol. (2022) 20:194–203.e1. doi: 10.1016/j.cgh.2020.08.045, PMID: 32835845 PMC8210475

[44] Darr U Khan Z Baig MA Khan MA Chakinala RC Solanki S. Burden of mental illness in hospitalized patients of liver cirrhosis: a Nationwide analysis from the National Inpatient Sample: 983. Official J Am Coll Gastroenterol. (2018) 113:S554–5. doi: 10.14309/00000434-201810001-00983

[45] Abureesh M Alkhayyat M Abualnadi I Badran R Henneberry JD Sadiq W. Epidemiology of depressive disorders in patients with liver cirrhosis: a population-based study in the United States. Prim Care Companion CNS Disord. (2022) 24:20m02889. doi: 10.4088/PCC.20m02889 35026872

[46] Shaffer LR Kaplan DE Taddei TH Mahmud N. The association between mental illness and all-cause mortality in patients with cirrhosis: a veterans affairs retrospective cohort study. Hepatol Commun. (2023) 7:e0129. doi: 10.1097/HC9.0000000000000129, PMID: 36996031 PMC10069831

[47] Rush AJ Trivedi MH Wisniewski SR Nierenberg AA Stewart JW Warden D. Acute and longer-term outcomes in depressed outpatients requiring one or several treatment steps: a STAR*D report. Am J Psychiatry. (2006) 163:1905–17. doi: 10.1176/ajp.2006.163.11.1905 17074942

[48] Cotter TG Beresford T. Treatment of mental health in patients with chronic liver disease. Clin Liver Dis (Hoboken). (2022) 20:57–60. doi: 10.1002/cld.1200, PMID: 36033423 PMC9405486

[49] Menon V Ransing R Praharaj SK. Management of Psychiatric Disorders in patients with hepatic and gastrointestinal diseases. Indian J Psychiatr. (2023) 64:S379–93. doi: 10.4103/indianjpsychiatry.indianjpsychiatry_18_22, PMID: 35602369 PMC9122174

[50] National Institute of Diabetes and Digestive and Kidney Diseases. LiverTox: clinical and research information on drug-induced liver injury [internet]. Bethesda, MD: National Institute of Diabetes and Digestive and Kidney Diseases. (2012). Available at: https://www.ncbi.nlm.nih.gov/books/NBK548752/ (Accessed April 8, 2020). 31643176

[51] National Institute of Diabetes and Digestive and Kidney Diseases. LiverTox: Clinical and research information on drug-induced liver injury [internet]. Bethesda (MD): National Institute of Diabetes and Digestive and Kidney Diseases; (2012). Available at: https://www.ncbi.nlm.nih.gov/books/NBK548836/ (Accessed Sepember 11, 2017). 31643176

[52] Kalaitzakis E. Gastrointestinal dysfunction in liver cirrhosis. World J Gastroenterol. (2014) 20:14686–95. doi: 10.3748/wjg.v20.i40.14686, PMID: 25356031 PMC4209534

[53] Fritz E Hammer J. Gastrointestinal symptoms in patients with liver cirrhosis are linked to impaired quality of life and psychological distress. Eur J Gastroenterol Hepatol. (2009) 21:460–5. doi: 10.1097/MEG.0b013e328318ed19, PMID: 19382343

[54] Theocharidou E Dhar A Patch D. Gastrointestinal motility disorders and their clinical implications in cirrhosis. Gastroenterol Res Pract. (2017) 2017:8270310–6. doi: 10.1155/2017/8270310, PMID: 28584525 PMC5444003

[55] Olson JC Saeian K. Gastrointestinal issues in liver disease. Crit Care Clin. (2016) 32:371–84. doi: 10.1016/j.ccc.2016.03.007 27339677

[56] Wang P Zhang YJ Li YR Liu XM Lv SY Xia XY. A correlation between gastrointestinal dysfunction and cirrhosis severity. Medicine (Baltimore). (2018) 97:e12070. doi: 10.1097/MD.0000000000012070, PMID: 30212936 PMC6156071

[57] Black CJ Drossman DA Talley NJ Ruddy J Ford AC. Functional gastrointestinal disorders: advances in understanding and management. Lancet. (2020) 396:1664–74. doi: 10.1016/S0140-6736(20)32115-2 33049221

[58] Duffy M Boggiano VL Ganesh R Mueller M. Functional gastrointestinal disorders. Prim Care. (2023) 50:429–46. doi: 10.1016/j.pop.2023.03.006 37516512

[59] Fikree A Byrne P. Management of functional gastrointestinal disorders. Clin Med (Lond). (2021) 21:44–52. doi: 10.7861/clinmed.2020-0980, PMID: 33479067 PMC7850201

[60] Samiullah S Malone M Waheed A. Functional gastrointestinal disorders: approach to patients with functional gastrointestinal disorders. FP Essent. (2018) 466:11–3. PMID: 29528204

[61] Neong SF Billington EO Congly SE. Sexual dysfunction and sex hormone abnormalities in patients with cirrhosis: review of pathogenesis and management. Hepatology. (2019) 69:2683–95. doi: 10.1002/hep.30359, PMID: 30468515

[62] Jagdish RK. Sexual dysfunctions and their treatment in liver diseases. World J Hepatol. (2022) 14:1530–40. doi: 10.4254/wjh.v14.i8.1530, PMID: 36157870 PMC9453461

[63] Burra P Germani G Masier A de Martin E Gambato M Salonia A. Sexual dysfunction in chronic liver disease: is liver transplantation an effective cure? Transplantation. (2010) 89:1425–9. doi: 10.1097/TP.0b013e3181e1f1f6, PMID: 20463637

[64] Durazzo M Premoli A Di Bisceglie C Bo S Ghigo E Manieri C. Male sexual disturbances in liver diseases: what do we know? J Endocrinol Investig. (2010) 33:501–5. doi: 10.1007/BF03346632, PMID: 20671409

[65] Darmadi D Pakpahan C Ruslie RH Amanda B Ibrahim R. The sex life of male patients with cirrhosis and its organic factors: what we have got so far? PLoS One. (2023) 18:e0280915. doi: 10.1371/journal.pone.0280915, PMID: 36730272 PMC9894452

[66] Rogal SS Beste LA Youk A Fine MJ Ketterer B Zhang H. Characteristics of opioid prescriptions to veterans with cirrhosis. Clin Gastroenterol Hepatol. (2019) 17:1165–1174.e3. doi: 10.1016/j.cgh.2018.10.021, PMID: 30342261 PMC8108399

[67] Holman A Parikh ND Zhao Z Nikirk S Clauw DJ Williams DA. Association between widespread pain and associated symptoms in patients with cirrhosis. Hepatol Commun. (2023) 7:e0120. Published April 14, 2023. doi: 10.1097/HC9.0000000000000120 37058114 PMC10109455

[68] Holman A Parikh N Clauw DJ Williams DA Tapper EB. Contemporary management of pain in cirrhosis: toward precision therapy for pain. Hepatology. (2023) 77:290–304. doi: 10.1002/hep.32598, PMID: 35665522 PMC9970025

[69] Fitzcharles MA Cohen SP Clauw DJ Littlejohn G Usui C Häuser W. Nociplastic pain: towards an understanding of prevalent pain conditions. Lancet. (2021) 397:2098–110. doi: 10.1016/S0140-6736(21)00392-5, PMID: 34062144

[70] Nijs J Lahousse A Kapreli E Bilika P Saraçoğlu İ Malfliet A. Nociplastic pain criteria or recognition of central sensitization? Pain phenotyping in the past, present and future. J Clin Med. (2021) 10:3203. doi: 10.3390/jcm10153203, PMID: 34361986 PMC8347369

[71] Cohen SP Vase L Hooten WM. Chronic pain: an update on burden, best practices, and new advances. Lancet. (2021) 397:2082–97. doi: 10.1016/S0140-6736(21)00393-7, PMID: 34062143

[72] Bułdyś K Górnicki T Kałka D Szuster E Biernikiewicz M Markuszewski L. What do we know about Nociplastic pain? Healthcare (Basel). (2023) 11:1794. doi: 10.3390/healthcare11121794, PMID: 37372912 PMC10298569

[73] Coghill RC. The distributed nociceptive system: a framework for understanding pain. Trends Neurosci. (2020) 43:780–94. doi: 10.1016/j.tins.2020.07.004, PMID: 32800534 PMC7530033

[74] Fernández-de-Las-Peñas C Nijs J Neblett R Polli A Moens M Goudman L. Phenotyping post-COVID pain as a nociceptive, neuropathic, or nociplastic pain condition. Biomedicines. (2022) 10:2562. doi: 10.3390/biomedicines10102562, PMID: 36289827 PMC9599440

[75] Afridi B Khan H Akkol EK Aschner M. Pain perception and management: where do we stand? Curr Mol Pharmacol. (2021) 14:678–88. doi: 10.2174/1874467213666200611142438, PMID: 32525788 PMC7728656

[76] Finnerup NB Kuner R Jensen TS. Neuropathic pain: from mechanisms to treatment. Physiol Rev. (2021) 101:259–301. doi: 10.1152/physrev.00045.2019 32584191

[77] Szok D Tajti J Nyári A Vécsei L. Therapeutic approaches for peripheral and central neuropathic pain. Behav Neurol. (2019) 2019:8685954. doi: 10.1155/2019/8685954 31871494 PMC6906810

[78] Bannister K Sachau J Baron R Dickenson AH. Neuropathic pain: mechanism-based therapeutics. Annu Rev Pharmacol Toxicol. (2020) 60:257–74. doi: 10.1146/annurev-pharmtox-010818-021524 31914896

[79] Bates D Schultheis BC Hanes MC Jolly SM Chakravarthy KV Deer TR. A comprehensive algorithm for Management of Neuropathic Pain [published correction appears in pain med. 2023;24(2):219]. Pain Med. (2019) 20:S2–S12. doi: 10.1093/pm/pnz075, PMID: 31152178 PMC6544553

[80] Klinge M Coppler T Liebschutz JM Dugum M Wassan A DiMartini A. The assessment and management of pain in cirrhosis. Curr Hepatol Rep. (2018) 17:42–51. doi: 10.1007/s11901-018-0389-7, PMID: 29552453 PMC5849403

[81] Chandok N Watt KD. Pain management in the cirrhotic patient: the clinical challenge. Mayo Clin Proc. (2010) 85:451–8. doi: 10.4065/mcp.2009.0534, PMID: 20357277 PMC2861975

[82] Tapper E Kanwal F Asrani S Ho C Ovchinsky N Poterucha J. Patient reported outcomes in cirrhosis: a scoping review of the literature. Hepatology (Baltimore, Md). (2017) 67:2375–83. doi: 10.1002/hep.29756 29272043

[83] Barakji J Korang SK Feinberg JB Maagaard M Mathiesen O Gluud C. Tramadol for chronic pain in adults: protocol for a systematic review with meta-analysis and trial sequential analysis of randomised clinical trials. Syst Rev. (2023) 12:145. doi: 10.1186/s13643-023-02307-0, PMID: 37608394 PMC10463795

[84] Ojeda A Moreno LA. Pain management in patients with liver cirrhosis. Gastroenterol Hepatol. (2014) 37:35–45. doi: 10.1016/j.gastrohep.2013.05.007, PMID: 24309482

[85] Rakoski M Goyal P Spencer-Safier M Weissman J Mohr G Volk M. Pain management in patients with cirrhosis. Clin Liver Dis (Hoboken). (2018) 11:135–40. doi: 10.1002/cld.711, PMID: 30992804 PMC6385960

[86] Al Aboud AM Syed HA Zito PM. Alopecia. In: StatPearls [Internet]. Treasure Island, FL: StatPearls Publishing; (2024). Available at: https://www.ncbi.nlm.nih.gov/books/NBK538178/ (Accessed February 26, 2024).

[87] Alessandrini A Bruni F Piraccini BM Starace M. Common causes of hair loss - clinical manifestations, trichoscopy and therapy. J Eur Acad Dermatol Venereol. (2021) 35:629–40. doi: 10.1111/jdv.17079, PMID: 33290611

[88] Bhandari A Mahajan R. Skin changes in cirrhosis. J Clin Exp Hepatol. (2022) 12:1215–24. doi: 10.1016/j.jceh.2021.12.013, PMID: 35814509 PMC9257870

[89] Liu Y Zhao Y Gao X Liu J Ji F Hsu YC. Recognizing skin conditions in patients with cirrhosis: a narrative review. Ann Med. (2022) 54:3016–28. doi: 10.1080/07853890.2022.2138961, PMID: 36308406 PMC9629063

[90] Patel AD Katz K Gordon KB. Cutaneous manifestations of chronic liver disease. Clin Liver Dis. (2020) 24:351–60. doi: 10.1016/j.cld.2020.04.003 32620276

[91] Godara SK Thappa DM Pottakkatt B Hamide A Barath J Munisamy M. Cutaneous manifestations in disorders of hepatobiliary system. Indian Dermatol Online J. (2017) 8:9–15. doi: 10.4103/2229-5178.198760, PMID: 28217465 PMC5297287

[92] Koulaouzidis A Bhat S Moschos J. Skin manifestations of liver diseases. Ann Hepatol. (2007) 6:181–4. doi: 10.1016/S1665-2681(19)31926-X 17786146

[93] Satapathy SK Bernstein D. Dermatologic disorders and the liver. Clin Liver Dis. (2011) 15:165–82. doi: 10.1016/j.cld.2010.09.001 21111999

[94] Lepe K Syed HA Zito PM. Alopecia areata. In: StatPearls [Internet] Treasure Island, FL: StatPearls Publishing; (2024). Available at: https://www.ncbi.nlm.nih.gov/books/NBK537000/ (Accessed February 8, 2024). 30725685

[95] Phillips TG Slomiany WP Allison R. Hair loss: common causes and treatment. Am Fam Physician. (2017) 96:371–8. PMID: 28925637

[96] Mannu GS Sudul M Bettencourt-Silva JH Tsoti SM Cunnick G Ahmed SF. Role of tamoxifen in idiopathic gynecomastia: a 10-year prospective cohort study. Breast J. (2018) 24:1043–5. doi: 10.1111/tbj.13080, PMID: 30079473

[97] Swerdloff RS Ng JCM. Gynecomastia: etiology, diagnosis, and treatment. In: Feingold KR Anawalt B Blackman MR, et al., editors. Endotext [Internet]. South Dartmouth, MA: MDText.com, Inc.; (2000). Available at: https://www.ncbi.nlm.nih.gov/books/NBK279105/ (Accessed January 6, 2023).

[98] Thawabteh AM Jibreen A Karaman D Thawabteh A Karaman R. Skin pigmentation types, causes and treatment-a review. Molecules. (2023) 28:4839. doi: 10.3390/molecules28124839, PMID: 37375394 PMC10304091

[99] Masub N Khachemoune A. Cosmetic skin lightening use and side effects. J Dermatolog Treat. (2022) 33:1287–92. doi: 10.1080/09546634.2020.1845597 33135510

[100] Davids LM Van Wyk JC Khumalo NP. Intravenous glutathione for skin lightening: inadequate safety data. S Afr Med J. (2016) 106:782–6. doi: 10.7196/SAMJ.2016.v106i8.10878, PMID: 27499402

